# Mechanochemical Polyoxometalate Super-Reduction with Lithium Metal

**DOI:** 10.1021/jacs.4c09998

**Published:** 2024-09-10

**Authors:** Magda Pascual-Borràs, Elisabetta Arca, Hirofumi Yoshikawa, Thomas Penfold, Paul G. Waddell, R. John Errington

**Affiliations:** 1 NUPOM Lab, Chemistry, School of Natural & Environmental Sciences, Newcastle University, NE1 7RU Newcastle Upon Tyne, U.K.; 2 School of Mathematics, Statistics and Physics, Newcastle University, NE1 7RU Newcastle Upon Tyne, U.K.; 3 Department of Materials Science, Kwansei Gakuin University, Sanda, Hyogo 669-1330, Japan

## Abstract

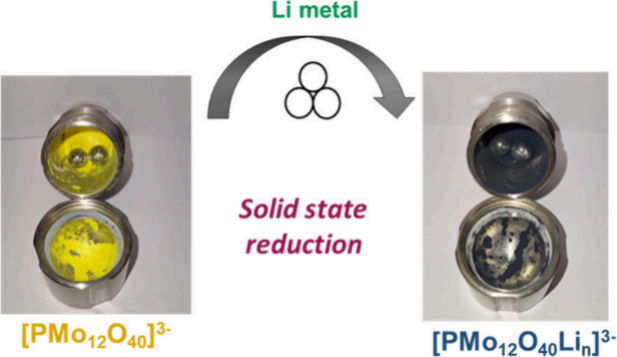

In this first systematic investigation of mechanochemical polyoxometalate (POM) reduction, (TBA)_3_[PMo_12_O_40_] was reacted with *n* equiv of lithium metal (*n* = 1–24) to generate **PMo**_**12**_**/*n*** products which were shown to be mixtures of electron-rich **PMo**_**12**_**Li**_*x*_ species. FTIR analysis revealed the lengthening/weakening of terminal Mo=O bonds with increasing levels of reduction, while EXAFS spectra indicated the onset of Mo–Mo bond formation at *n* ∼ 8 and a significant structural change at *n* > 12. Successive Mo^VI^ reductions were monitored by XANES and XPS, and at *n* = 24, results were consistent with the formation of at least one Mo^IV^–Mo^IV^ bonded {Mo^IV^_3_} triad together with Mo^V^. Upon dissolution, the **PMo**_**12**_**Li**_***x***_ species present in the solid **PMo**_**12**_**/*n*** products undergo electron exchange and single-peak ^31^P NMR spectra were observed for *n* = 1–12. For *n* ≥ 16, changes in solid state and solution ^31^P NMR spectra coincided with the emergence of features in the UV–vis spectra associated with Mo^V^–Mo^V^ and {Mo^IV^_3_} bonding in an ε-Keggin structure. Bonding between {Li(NCMe)}^+^ and 2-electron-reduced **PMo**_**12**_ in (TBA)_4_[PMo_12_O_40_{Li(NCMe)}] suggests that super-reduction gives rise to more extensive Li–O bonding that ultimately causes lithium-oxide-promoted TBA cation decomposition and POM degradation, which might explain the appearance of XPS peaks for Mo_2_C at *n* ≥ 16. This work has revealed some of the complex, unexplored chemistry of super-reduced POMs and establishes a new, solvent-free approach in the search for a better fundamental understanding of the electronic properties and reactivity of electron-rich nanoscale metal oxides.

## Introduction

Polyoxometalates (POMs), the polynuclear oxoanions of the groups 5 and 6 transition metals (V, Nb, Ta, Mo, W), adopt a wide range of structures with dimensions ranging from a few ångströms up to several nanometers and compositions that can include elements from across most of the periodic table.^1−3^ The most commonly studied POM structures are based on Keggin-type anions, i.e., [EM_12_O_40_]^*n*−^, where E is a central heteroatom within the metal oxide framework, as first observed for H_3_[PW_12_O_40_] and characterized by powder X-ray diffraction in 1933.^4^

Much of the interest in POMs derives from their redox activity, and they are often regarded as electron reservoirs due to an ability to reversibly accept multiple electrons; as such, they are excellent models for mechanistic studies of electrochemically active metal oxides. The initial reduced states of Keggin-type POMs, known as “heterpoly blues” because of the deep-blue colors associated with intervalence charge transfer transitions, have been widely studied,^5^ and the phosphomolybdate blue arising from reduction of [PMo_12_O_40_]^3–^ was first reported in 1783.^6^

Theoretical and experimental studies of reduced Keggin-type POMs have highlighted the effects of electron–electron interactions on the bonding and physical properties of these nanometre sized metal oxides, e.g., bipolaron formation, magnetism, vibronic coupling, and metal–metal bond formation,^7−11^ and mechanistic details of electrochemical reduction have been investigated,^12^ but the chemistry of electron-rich, super-reduced systems has received comparatively little attention.^13^

The multielectron capacity of Keggin-type POMs, which was first demonstrated in the seminal electrochemical studies of [H_2_W_12_O_40_]^6–^ by Launay,^14^ has stimulated much interest in their use as energy storage materials.^13,15,16^ On the basis of operando XAFS measurements, Awaga and co-workers suggested that [PMo_12_O_40_]^3–^ underwent reversible 24-electron reduction when incorporated as a cathode material in a lithium ion battery.^17^ Subsequent DFT and molecular dynamics calculations provided support for the suggestion that all 12 Mo^VI^ sites were reduced to Mo^IV^ with localization of the added 5d electrons in the metal–metal bonds of four {Mo^IV^}_3_ cluster units within the reduced Keggin structure.^18^ The formation of metal–metal bonded {W^IV^_3_} triads upon six-electron reduction of tungstates [H_2_W_12_O_40_]^6–^, [BW_12_O_40_]^5–^, and [SiW_12_O_40_]^4–^ has been established by ^183^W NMR spectroscopy and X-ray crystallography.^7,8,11,19^ In contrast, no analogous structures have been obtained by reduction of α-Keggin molybdates, although aqueous oxidation of trinuclear [Mo_3_O_4_(H_2_O)_9_]^4+^ gave a β-Keggin molybdate containing two metal–metal bonded {Mo^IV^_3_} triads and a capping {Mo^VI^O_2_}^2+^ fragment with an average Mo^IV^–Mo^IV^ single bond distance of 2.5 Å, and longer nonbonding Mo^IV^–Mo^IV^, Mo^IV^–Mo^VI^ and Mo^VI^–Mo^VI^ distances for MoOMo linkages averaging 3.8, 3.7, and 3.4 Å respectively.^20^ More typically, multiply reduced Keggin-type molybdates adopt ε-type structures such as ε-[PMo^V^_8_Mo^VI^_4_O_40_H_3_]^8–^ and ε-[H_2_Mo^V^_12_O_30_(μ-OH)_10_]^8–^, which contain isolated Mo^V^–Mo^V^ bonds and capping M^2+^ or M^3+^ cations.^21−24^ The average Mo^V^–Mo^V^ single bond distance in the ε-{PMo_12_O_40_Zn_4_} anions is about 2.6 Å. Most recently, Falaise and co-workers reported the electrochemical synthesis of six-electron reduced [SiMoW_11_O_37_(H_2_O)_3_]^4–^ containing a {Mo^IV^W^IV^_2_} cluster with the first heterometallic Mo–W bond to be observed in a Keggin-type POM,^9^ while Xu and co-workers obtained heteroatom-derivatized, 14-electron-reduced ε-Keggin type molybdates containing two {Mo^IV^_3_} triads and a Mo^V^–Mo^V^ bond from [Mo^IV^_3_O_2_(O_2_CCH_3_)_6_(H_2_O)_3_]^2+^.^21b^

Various reports have described charge storage and energy conversion materials based on electron-rich POMs generated under electrochemical conditions,^25,26^ but the rational and systematic formation of reduced/super-reduced POMs by controlled *chemical* reduction either in the solid state or in solution has scarcely been investigated.^27−30^ Structurally characterized examples of reduced Keggin-type POMs have been obtained by electrolysis or from hydrothermal reactions in which the redox chemistry is often not fully understood.^20,31,32^

Mechanochemistry, i.e., the use of mechanical forces to initiate chemical reactions, has expanded during the past decade due to potential advantages that include solvent-free procedures, easy handling, high conversion rates, and minimal waste production.^33−36^ In addition, mechanochemistry has been shown to provide access to compounds that are otherwise difficult or impossible to obtain in solution.^37−40^ The use of mechanochemistry for either synthesis or reactivity studies in POM chemistry has hardly been explored, with only a few reports describing the synthesis of molybdates,^41^ cation exchange,^42−44^ or POM encapsulation within metal–organic frameworks.^45^ In this regard, the use of mechanochemistry in a systematic study of the stepwise reduction of POMs represents a new approach in the search for a better fundamental understanding of the electronic properties and reactivity of electron-rich nanoscale metal oxides. Herein, we report the first stepwise mechanochemical reduction of (TBA)_3_[PMo_12_O_40_], (TBA)_3_**PMo**_**12**_, and the observation of spectroscopic features associated with the onset of Mo–Mo bond formation in the solid state. Products have been analyzed by nuclear magnetic resonance (NMR), ultraviolet–visible (UV–vis) and Fourier transform infrared (FTIR) spectroscopy, X-ray photoelectron spectroscopy (XPS), X-ray absorption spectroscopy (XAS), and electrochemical methods in order to monitor the formation and investigate the nature of the resulting reduced and “super-reduced” POMs. Details of the experimental procedures and computational methods are presented in the Supporting Information.

## Results

**Scheme 1:**
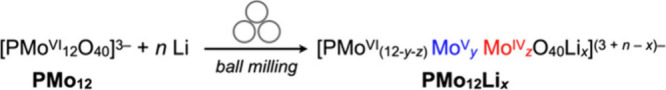
Mechanochemical Reduction of **PMo**_**12**_ To Give Solid Products **PMo**_**12**_**/*n*** Containing Reduced Species **PMo**_**12**_**Li**_***x***_

(TBA)_3_**PMo**_**12**_ was ball-milled with from 1 to 24 mol equiv of lithium metal under argon at 25–30 Hz for 60 min (see Supporting Information) in an attempt to access reduced derivatives of **PMo**_**12**_ from **PMo**_**12**_**(I)** to **PMo**_**12**_**(XXIV)** where Roman numerals indicate the number of electrons added to the **PMo**_**12**_ framework. Solid products **PMo**_**12**_**/*n*** were obtained, where *n* represents the number of mole equivalents of Li used in the reaction, and the reduced species present in these products are represented by **PMo**_**12**_**Li**_***x***_ (Scheme 1).

In order to mitigate excessive heat generation, products for *n* = 16, 20, or 24 were obtained stepwise by subsequent addition of 4, 8, or 12 mol equiv of Li respectively to the **PMo**_**12**_**/12** product. After milling, the products ranged in color from dark blue to brown, consistent with the intervalence charge transfer in reduced “heteropoly blues” and possible formation of metal–metal bonded “heteropoly browns”.^46,47^ It is worth noting that the transition from heteropoly blues to heteropoly browns containing metal–metal bonded {M^IV^_3_} units has only been established crystallographically for tungstates containing six-electron-reduced {W^IV^_3_} or {Mo^IV^W^IV^_2_} triads, although formation of more highly reduced Keggin-type tungstates with from two to four {W^IV^_3_} triads has been proposed from electrochemical studies.^14,46,47^ Spectroscopic and electrochemical studies of the **PMo**_**12**_**/*n*** products and solutions obtained from these solids are described below.

### Solid State Characterization

#### NMR Spectroscopy

**Figure 1:**
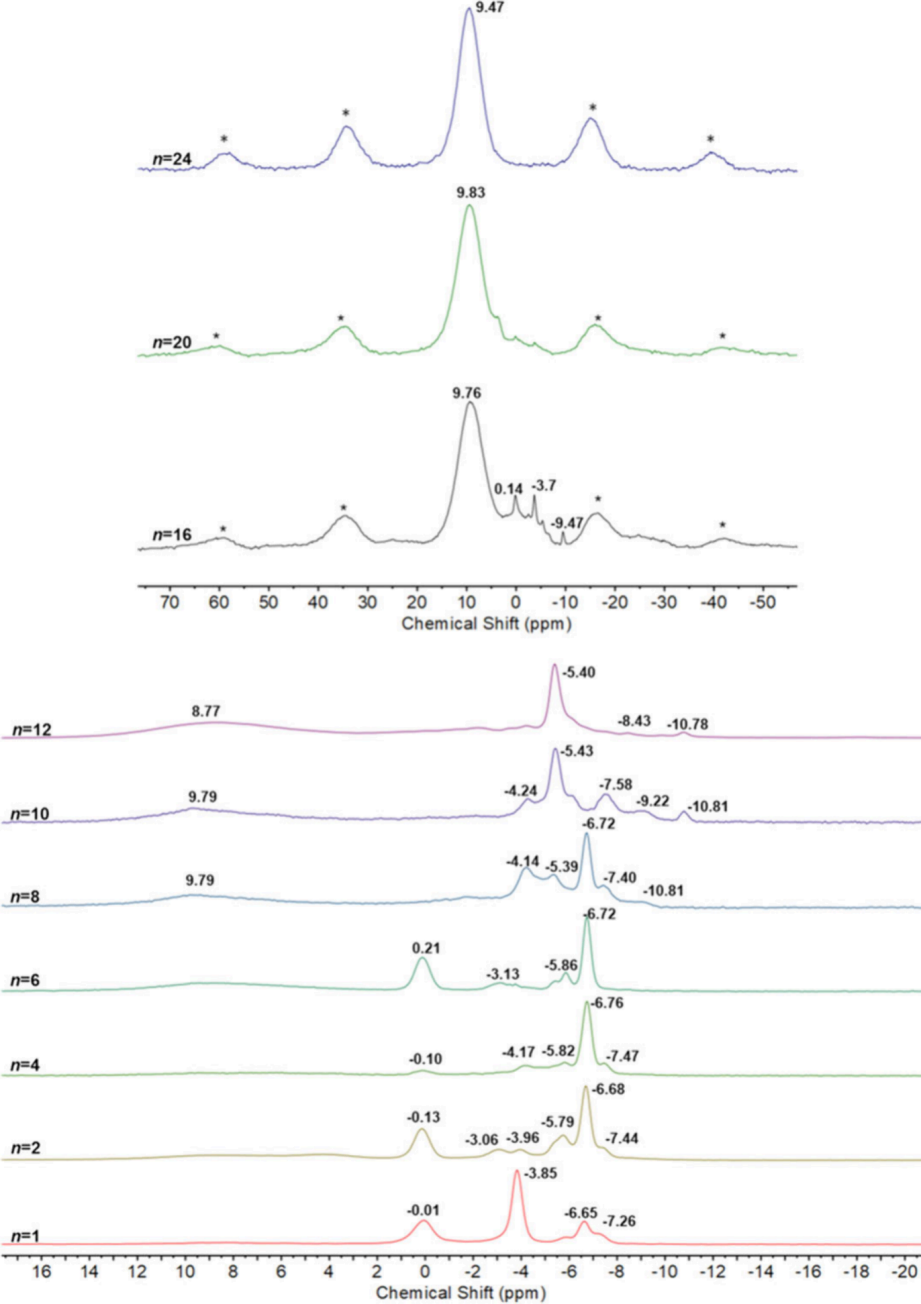
^31^P MAS NMR spectra of **PMo**_**12**_**/*n*** solids (*n* = 0–24). Peaks marked with an asterisk are spinning sidebands.

Powder XRD patterns for the solid samples were broad and ill-defined, indicating amorphous materials, but the solid state ^31^P MAS NMR spectra of the **PMo**_**12**_**/*n*** products in Figure 1 were more informative and show that different mixtures of species with varying degrees of reduction were obtained as the number of mole equivalents (*n*) of Li was increased. At *n* = 1, the peaks at −3.85 and −0.01 ppm are due to **PMo**_**12**_ and one-electron reduced **PMo**_**12**_**(I)** respectively, while the peak at −6.65 is assigned to **PMo**_**12**_**(II)**. The relative amounts of the different reduced species are seen to vary with increasing *n* until, at *n* = 8, a broad peak at ∼9 ppm appears. At *n* = 12, two major broad peaks are present, and at *n* = 24, only one broad peak at 9.47 ppm dominates.

#### IR Spectroscopy

**Figure 2:**
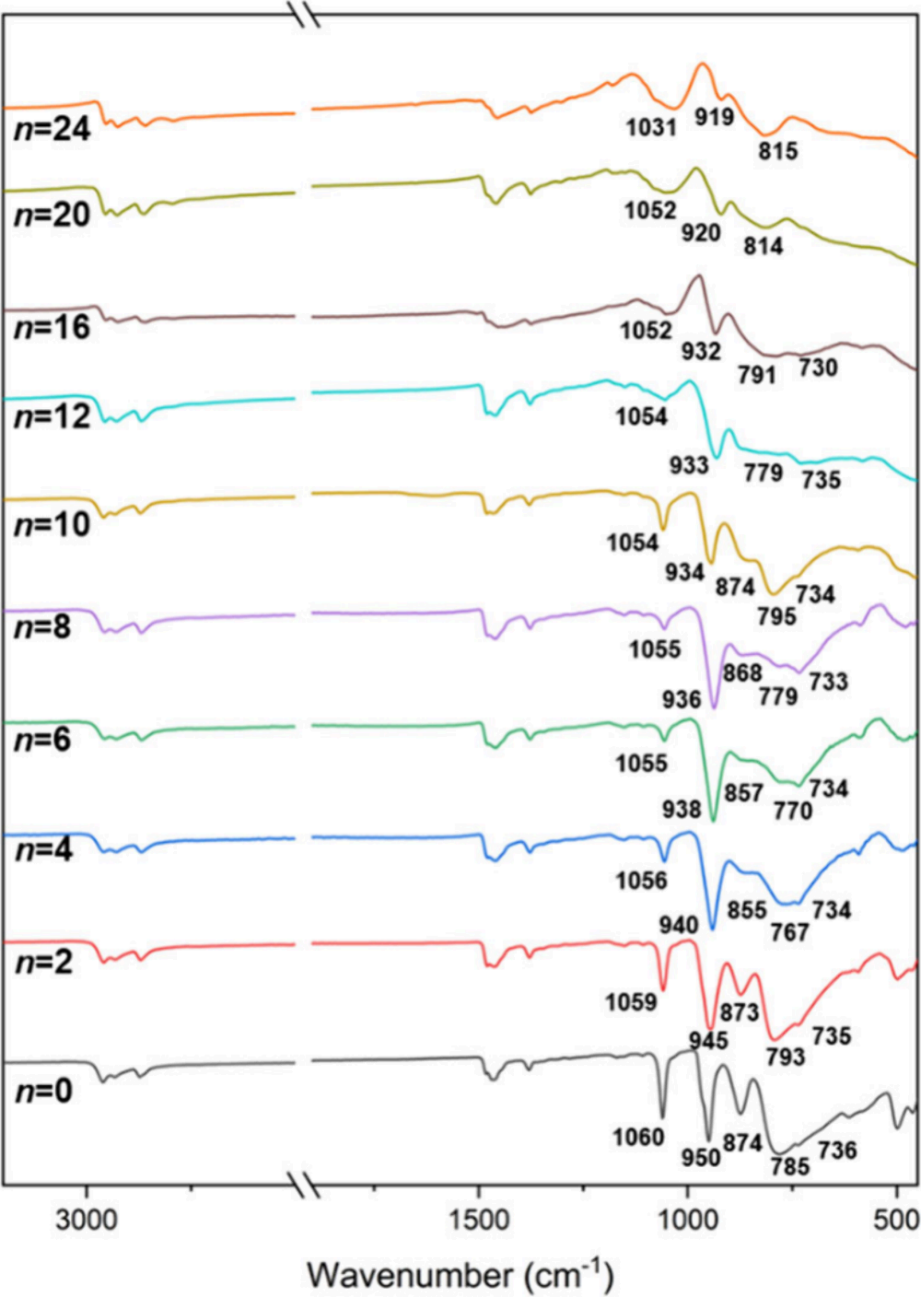
ATR FTIR spectra of **PMo**_**12**_**/*n*** solids (*n* = 0–24).

The characteristic vibrational absorptions for **PMo**_**12**_ have been studied both theoretically and experimentally, and the effects of different cations and structural rearrangement have been discussed.^48,49^ In situ spectroelectrochemical studies (IR and UV–vis) of **PMo**_**12**_ by up to four electrons have also been reported.^50^ ATR FTIR spectra of **PMo**_**12**_ and **PMo**_**12**_**/*n*** products (*n* = 1–24) are shown in Figure 2, and several features and trends are evident. Bands due to ν(P–O) at 1060 cm^–1^, ν(Mo=O) at 950 cm^–1^, ν(Mo–O–Mo) at 874 cm^–1^ (for links between {Mo_3_O_13_} units with larger MoOMo angles), and ν(Mo–O–Mo) at 785 cm^–1^ (for MoOMo bridges with smaller angles within {Mo_3_O_13_} units) all shift to lower wavenumber with concomitant changes in peak widths and intensities as more electrons are added. The most dramatic changes are associated with ν(Mo=O) and are consistent with gradual weakening and loss of terminal Mo=O bonds.

#### X-ray Absorption Near-Edge Structure (XANES) Spectroscopy

**Figure 3:**
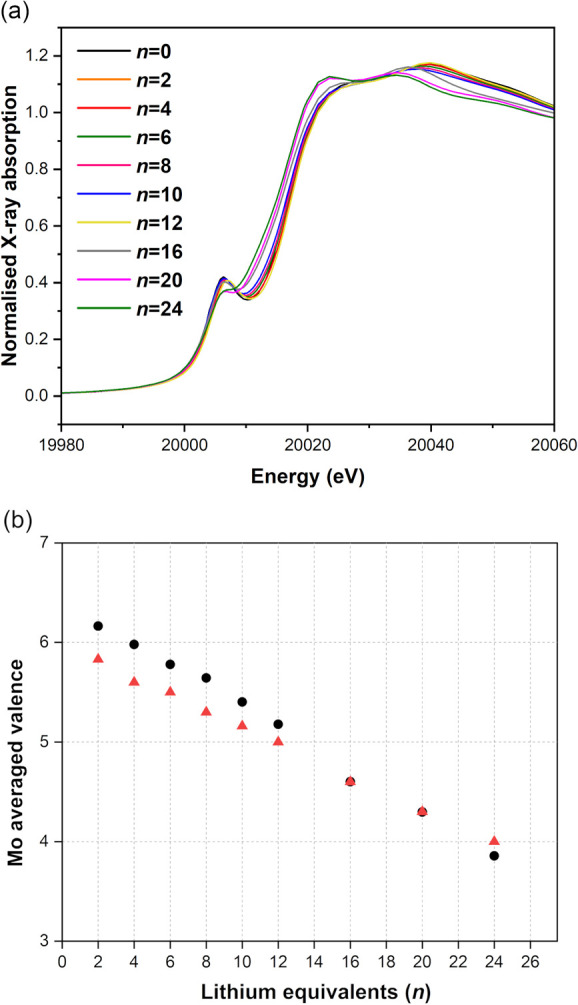
(a) Mo K-edge XANES spectra for **PMo**_**12**_**/*n*** products. (b) Observed (black circle) and expected (red triangle) averaged Mo valence in **PMo**_**12**_**/*n*** as a function of the number of mole-equivalents (*n*) of lithium used in the reaction.

XANES spectra for **PMo**_**12**_**/*n*** products at the Mo K-edge are presented in Figure 3a. The pre-edge peak is associated with a dipole-forbidden 4d ← 1s transition and gains intensity from 4d/5p orbital mixing due to deviations from a perfectly octahedral geometry which is directed primarily along the terminal Mo=O.^17,51,52^ The disappearance of this peak with the increasing amount of lithium added indicates the elimination of short Mo=O bonds at more highly reduced molybdenum sites, driving structural changes that cause the first coordination shell around the Mo absorbing atoms to more closely obey octahedral symmetry, precluding the 4d/5p mixing. Besides the pre-edge, these XANES spectra show additional key differences that have been previously highlighted in the computational studies of Penfold and co-workers,^51^ i.e., (1) a red shift of the absorption energy edge, (2) an increase in the white-line intensity, and (3) a loss of intensity at 20.05 keV.

The shift in the absorption edge is associated with the reduction of the Mo sites leading to a reduction of the binding energy of the core electrons.^53^ As both the increase in the white-line intensity and the loss of intensity at 20.05 keV occur above the absorption edge, these changes are associated with structural modifications occurring upon reduction. Given the previous analysis,^51^ these will be most strongly associated with changes in the Mo–O bond lengths and O–Mo–O bond angles. At more highly reduced states, the formation of Mo–Mo bond may also contribute; however, it is important to recognize that backscattering between heavier elements such as Mo tends to occur at high values of energy.

The Mo K-edge XANES absorption edge energies were used to determine the oxidation state of molybdenum. As previously reported for XANES analyses of other molybdenum-containing materials, the edge energy is defined as the point at which the intensity reaches 60% of the absorption peak maximum.^17,54−56^ Using this relation, the *average* Mo oxidation state in solid **PMo**_**12**_**/*n*** products was calculated and plotted against the number of equivalents of added lithium (*n*) as shown in Figure 3b. Experimental observations (black) are close to those expected (red) for uniform, gradual reduction, such that the average oxidation state in the product from reduction with 12 equiv of lithium is Mo^V^ (i.e., at this stage all Mo^VI^ sites have been reduced on average by one electron) and Mo^IV^ after reduction with 24 equiv of lithium.

#### Extended X-ray Absorption Fine Structure (EXAFS) Analysis

The **α-PMo**_**12**_ anion can be regarded as a tetrahedral arrangement of four {Mo_3_O_3_(μ-O)_3_(μ_3_-O)} triangular units linked through μ-O bridges and bonded via μ_3_-O to a central P atom. The anions are disordered in reported structures of the TBA salt, but we obtained the nondisordered X-ray crystal structure of the bis(triphenylphosphine)iminium (PPN) salt and details are given in Figure S1 and Tables S1 and S2 to provide reference bond lengths for analysis of EXAFS data. There are no cation–anion H–O contacts of <2.4 Å and the average bond distance for short terminal Mo=O is 1.68 Å, with longer (2.02 Å) and shorter (1.85 Å) bridging Mo–O_Mo_ due to bond length alternation within and between {Mo_3_} units. Mo–O_P_ bonds (2.43 Å) are *trans* to terminal Mo=O, while Mo–Mo distances within {Mo_3_} units (3.42 Å) are shorter than those between the {Mo_3_} units (3.70 Å). This is consistent with previously reported EXAFS analysis of solid (TBA)_3_**PMo**_**12**_ at 80 K, which gave a band in the Fourier transform spectrum for terminal Mo=O, two bands for Mo–O, and two bands for Mo–Mo.^57^ Similar EXAFS results were described for Cs_3–*x*_H_*x*_[PMo_12_O_40_] (*x* = 1, 3).^58^ The reduction-induced structural changes for **PMo**_**12**_**Li**_***x***_ species within the **PMo**_**12**_**/*n*** products were probed by EXAFS and the κ^3^χ(κ) functions for the Mo K-edge absorption spectra are depicted in Figure S2.

**Figure 4:**
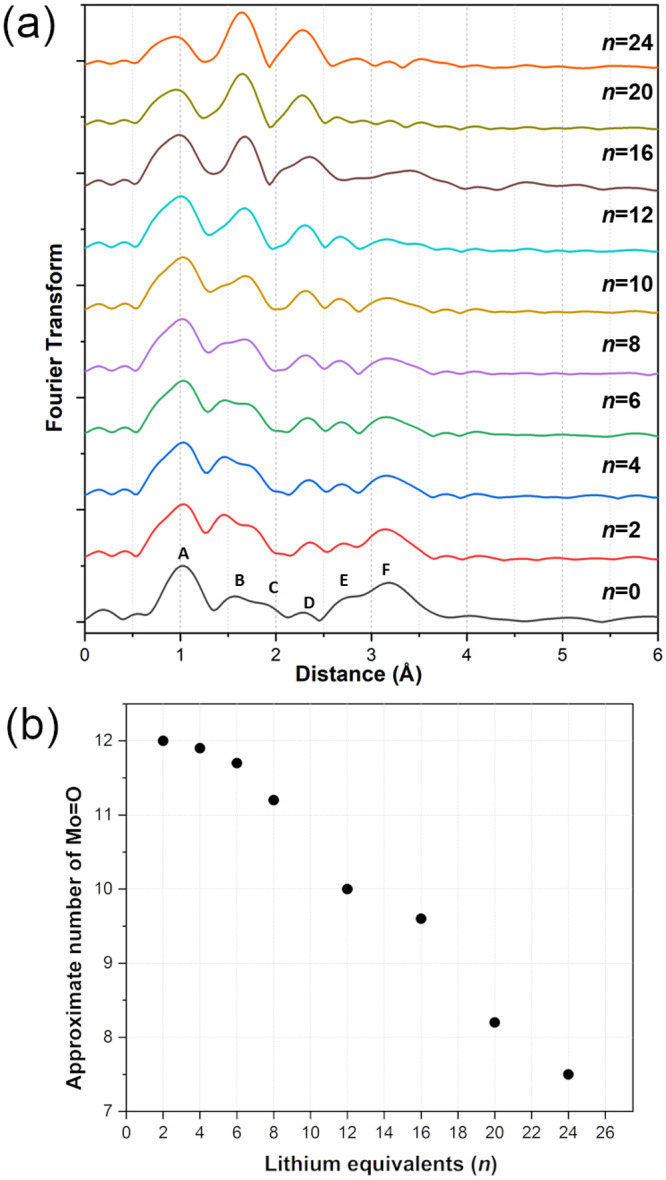
(a) Experimental Fourier transforms of the Mo *K* edge EXAFS spectra for **PMo**_**12**_**/*n*** products. (b) Number of terminal Mo=O bonds as a function of the number of mole-equivalents (*n*) of Li used in the reaction.

Fourier transforms of the κ^3^-weighted data for **PMo**_**12**_**/*n*** samples are shown in Figure 4a and the six bands evident for *n* = 0 can be assigned to Mo=O (A), Mo–O_Mo_ (B, C), M–O_P_ (D), and Mo–Mo (E, F). Bands E and F diminish with the increasing amounts of lithium, while bands B and D are significantly enhanced, and band A decreases in intensity (Table S3). The increase in the intensity of band B is due to the transformation of terminal Mo=O double bonds to Mo–O single bonds, presumably as a result of interactions with Li^+^, as also observed in FTIR and XANES spectra, while the greater intensity in the region of band D is due to the shortening of the Mo–Mo distance. At the highest reduction state, i.e., *n* = 24, the spectra show only three bands at 1 Å, 1.7 Å, and 2.3 Å, which are attributed to residual terminal Mo=O, Mo–O, and overlapping bands due to Mo–O_P_ and Mo–Mo (Mo^IV^–Mo^IV^ and possibly Mo^V^–Mo^V^). These results show that the Mo–Mo distances shorten significantly from those observed in the fully oxidized anion, confirming the formation of Mo–Mo bonds during mechanochemical reduction with lithium metal. It is notable that band A associated with short terminal Mo=O bonds decreases in intensity as the number of equivalents of lithium increases but does not disappear completely, in contrast to the operando electrochemical studies by Awaga and co-workers, in which this band was not observed for the discharged state of the cell, i.e. when the POM was fully reduced.^17^ The intensity of band A for the **PMo**_**12**_**/*n*** products was used to estimate the number of Mo=O bonds present at different degrees of reduction (Figure 4b). It can be seen that the overall number of Mo=O bonds decreases almost linearly as the number of Li equivalents (*n*) increases. However, at *n* = 24, when all of the Mo=O bonds are expected to be weakened and elongated, our results show that an average of approximately seven short terminal Mo=O bonds remain in the constituent **PMo**_**12**_**Li**_***x***_ species, most probably as Mo^V^=O. The correlation of terminal Mo=O bond lengthening with reduction to Mo^IV^ therefore suggests that only about half of the Mo^VI^ sites were reduced to Mo^IV^ in this reaction.

One of the challenges regarding the Fourier transform of EXAFS spectra is that for systems that contain many scattering paths of different atomic species and/or single and multiple scattering pathways that contribute to the same region of R-space, an unambiguous assignment of the peaks can be difficult. A further analysis of the EXAFS spectra for **PMo**_**12**_**/*n*** products can be achieved using a wavelet analysis,^59,60^ as presented in Figure S3. This shows that the Mo–O bands, which are the dominating pathways in the EXAFS spectra, merge upon reduction, indicating that the internal Mo–O bond lengths become less asymmetric when most of the Mo sites are reduced. The Mo–Mo band occurs at shorter distances and overlaps with the intense Mo–O band which, in agreement with recent computational modeling by Penfold and co-workers, highlights the challenge in accurately quantifying the formation Mo–Mo bonds during reduction.^51^

#### X-ray Photoelectron Spectroscopy (XPS)

XPS analysis of the **PMo**_**12**_**/*n*** products was carried out in an attempt to determine Mo valence states and compositions (Figure S4). The identification of a given chemical environment in these products is based on the relative position of core levels (ΔBE) of their constituent elements. This serves as a more accurate quantification than the attribution based on the absolute peak position. A previous study demonstrated that for a chemical compound, peaks for all the constituent elements will appear shifted by the same amount if effects such as charging, charge neutralization, change in the Fermi level position due to doping, etc. are in place. Thus, whereas the absolute peak position on the binding energy scale will vary, their separation (ΔBE) is a constant quantity.^61,62^ To establish the different ΔBE, average ΔBE for the Mo–O binding energy separation for the various Mo oxidation states were calculated from literature values as listed in Table S4. The Mo 3d spectra of **PMo**_**12**_**Li**_***x***_ species in the **PMo**_**12**_**/*n*** products (*n* = 0–24) are shown in Figure S4a. For *n* = 0, 2, 4, and 8, ΔBE_Mo–O_ indicates the presence of Mo^VI^ and Mo^V^,^63^ and it is evident from the results for (TBA)_3_[PMo_12_O_40_] (*n* = 0) that some reduction of Mo^VI^ to Mo^V^ occurs upon irradiation with X-rays.^64^ The O 1s spectra (Figure S4b) peaks are assigned to Mo–O and P–O, respectively. P 2p spectra show peaks corresponding to P–O bonding (Figure S4c).^65^ The peaks at C 1s can be assigned to C–C/C=C, C–N, and C–O (Figure S4d).^65−67^ The appearance of organic C–O environments at higher reduction states is most likely due to decomposition of the tetrabutylammonium cations. As *n* is increased to 10 and 12, extra peaks appear in Mo 3d spectrum at lower binding energies assigned to Mo^IV^.^66,68^ The O 1s spectra include the peaks for C–O bonds, which is consistent with the C–O peak suggested by the C 1s spectra. Moreover, at *n* = 16, 20, and 24, an extra Mo environment is present, which corresponds to previous reports of Mo^II^ in Mo_2_C.^66,69,70^ The Mo–C peak characteristic of Mo_2_C ^66,71^ is also apparent in the C 1s spectrum (Figure S4d). Li 1s spectra (Figure S4e) showed only one peak, indicative of similar environments for the Li^+^ ions,^72^ and it is worth noting that Li was not detected for samples where *n* = 2 and 4, possibly due to the low amounts present and sensitivity limits.

**Figure 5:**
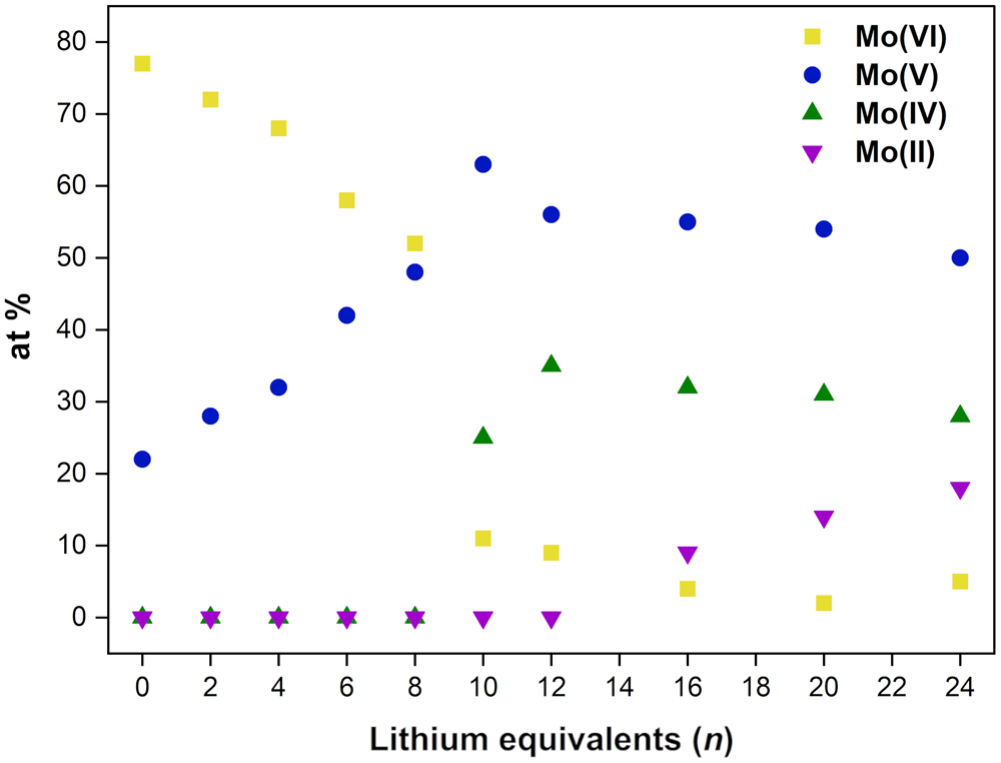
Atomic percentage of the different molybdenum environments present in **PMo**_**12**_**/*n*** products as a function of mole-equivalents (*n*) of lithium used in the reaction.

Figure 5 shows the atomic percentage of the various Mo oxidation states in **PMo**_**12**_**/*n*** products as a function of the number of mole equivalents of Li added in the reduction reactions. This was calculated as the ratio between the peak areas associated with each oxidation state. The reduction by X-rays of Mo^VI^ to Mo^V^ in **PMo**_**12**_**/*n*** products with lower values of *n* introduces uncertainties in the data, but the expected increase in Mo^V^ at the expense of Mo^VI^ is indeed observed as more Li is added until, at *n* = 10, there is evidence for the presence of Mo^IV^. For *n* > 10, the percentage of Mo^IV^ is fairly constant at around 30 atom %, which is consistent with the EXAFS result as in Figure 4b, while the amount of Mo^V^ remains at around 50–55 atom %. Given the equal amounts of Mo ^VI^ and Mo^V^ detected at *n* = 8, approximately 10% Mo^IV^ would be expected if all eight electrons are transferred to **PMo**_**12**_. The absence of Mo^IV^ at *n* = 8 may be attributed to a lack of sensitivity and the small amount of Mo^IV^ present. Interestingly, the amount of Mo^II^ attributed to Mo_2_C increases to about 18% at *n* = 24.

### Solution Characterization

#### NMR Spectroscopy

**Figure 6:**
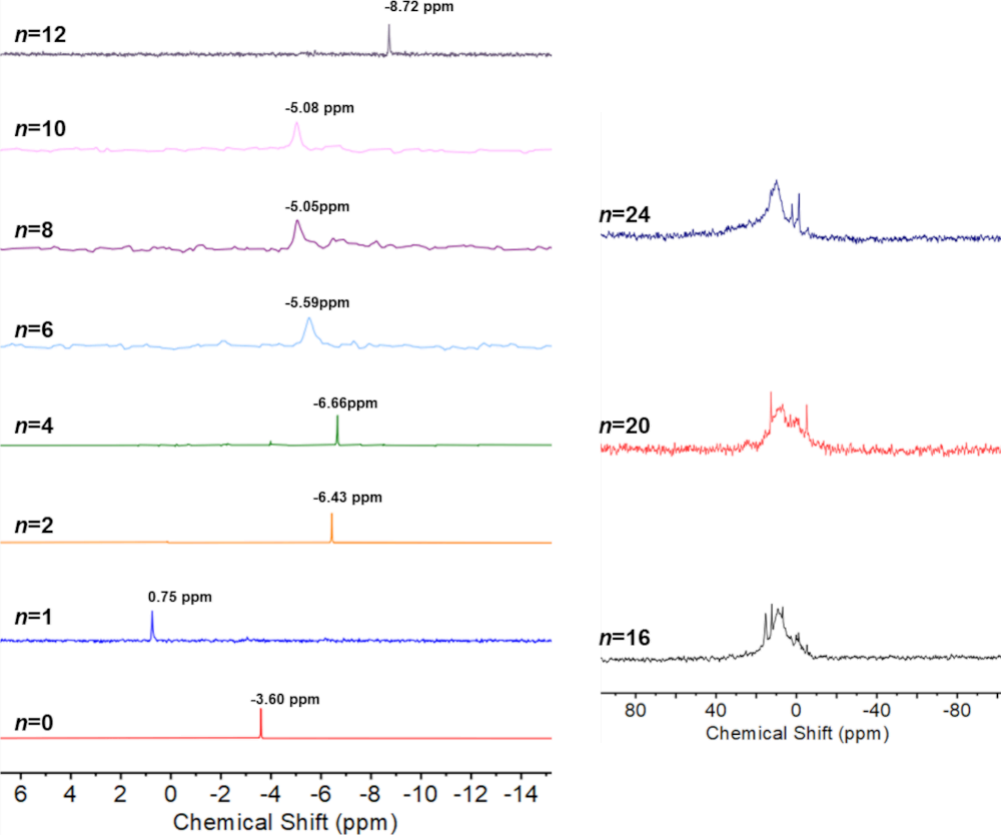
^31^P NMR spectra of **PMo**_**12**_ and **PMo**_**12**_**/*n*** products (*n* = 1–24) in a propylene carbonate solution.

Figure 6 shows ^31^P NMR spectra of solutions obtained by the addition of propylene carbonate to solid **PMo**_**12**_**/*n*** products. The more highly reduced materials with *n* = 16–24 were not totally soluble; therefore, spectra represent the more soluble components. In contrast to the solid-state spectra in Figure 1, the single peaks observed for dissolved **PMo**_**12**_**/*n*** products with *n* = 1–12 are suggestive of single **PMo**_**12**_**Li**_***x***_ species for each of these samples. Upon dissolution of **PMo**_**12**_**/1**, the single peak at 0.75 ppm, downfield of the peak for **PMo**_**12**_ at −3.6 ppm, is consistent with one-electron reduced **PMo**_**12**_**(I)** and shows that electron exchange between **PMo**_**12**_ and **PMo**_**12**_**(II)** present in the solid product, as shown by ^31^P MAS NMR, is rapid and efficient upon addition of a solvent. For solutions of **PMo**_**12**_**/*n*** with even values of *n*, peaks are observed upfield of the peak for **PMo**_**12**_ and the line broadening and gradual downfield shift for *n* = 6–10 may indicate relatively fast electron exchange between different species in solution. The spectrum for **PMo**_**12**_**/12** containing a single sharp upfield peak at −8.72 ppm appears to indicate a change in the nature of the solution species, and at higher degrees of reduction (*n* ≥ 16), spectra are characterized by very broad resonances shifted significantly downfield accompanied by a few minor sharp peaks. Solid state ^31^P NMR spectra of the soluble and insoluble components of the **PMo**_**12**_**/*n*** products (*n* = 12, 16, 20, 24) after extraction into MeCN, filtration, removal of the solvent from the filtrate, and drying both fractions showed the narrow peaks to be associated with more soluble components (Figure S5).

#### Electronic Spectroscopy

**Figure 7:**
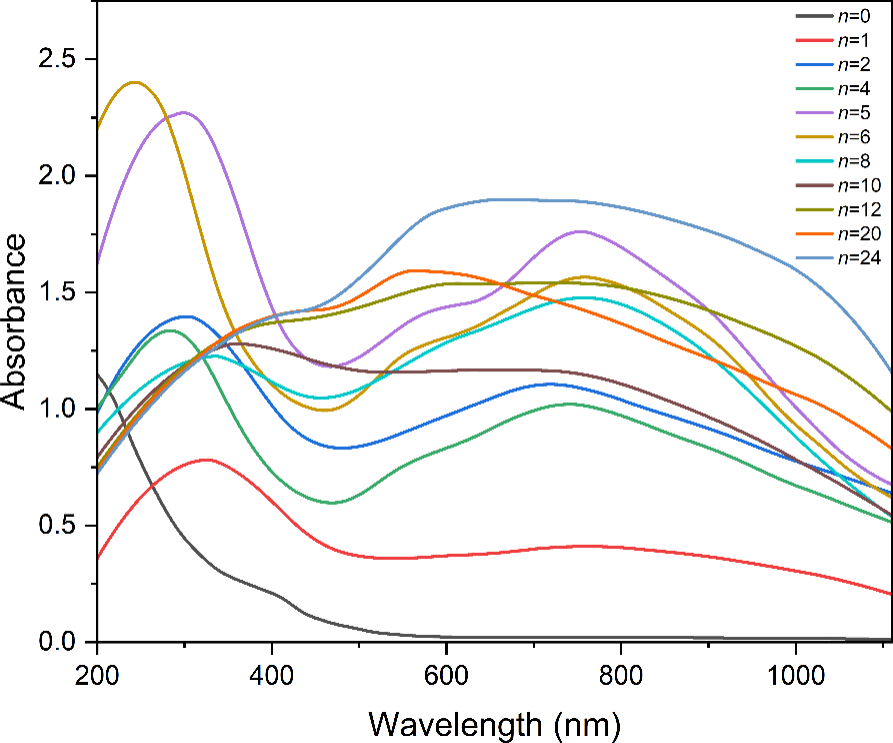
UV–vis spectra of **PMo**_**12**_ and **PMo**_**12**_**/*n*** reduction products dissolved in MeCN (0.2 mM).

Further insight into the solutions obtained from the reduced **PMo**_**12**_**/*n*** products in MeCN (*n* = 1–24) was provided by the UV–visible spectra shown in Figure 7. Spectra for *n* = 1, 2, and 4 are consistent with previous studies of electrochemically generated **PMo**_**12**_**(I)**, **PMo**_**12**_**(II)**, and **PMo**_**12**_**(IV)** in the absence of Li^+^ and are characterized by O_π_ → Mo_d_ charge transfer bands at ∼300 nm and intervalence bands at approximately 715–750 nm.^50^ Spectra for *n* = 6 and 8 feature more intense MLCT bands, with a significant shift to higher energy for *n* = 8, and the appearance of bands at 580 and 559 nm, respectively, as shoulders on intervalence bands centered at ∼750 nm. For *n* = 10–24, more extensive reduction and higher [Li^+^] leads to less intense MLCT bands shifted gradually to lower energy, with increasingly intense absorption at 550–600 nm and more expansive intervalence bands. Features in these spectra can be compared with electronic spectra of aqueous Mo–Mo bonded [Mo^V^_2_O_4_]^2+^ and [Mo^IV^_3_O_4_]^4+^ and related complexes, which are characterized by O_π_ → Mo_d_ MLCT bands at 200–250 nm for the Mo^V^ species and d–d bands at ∼400 nm for Mo^V^–Mo^V^ and 505–550 nm for Mo^IV^–Mo^IV^ bonded species.^73−75^ Interestingly, the expansive absorption for *n* = 24 is similar to those observed for the 14-electron reduced Mo^IV^_6_-ε-Keggin structures containing two {Mo^IV^_3_} triads and a Mo^V^–Mo^V^ bond reported by Xu and co-workers.^21b^

#### Electrochemistry

**Figure 8:**
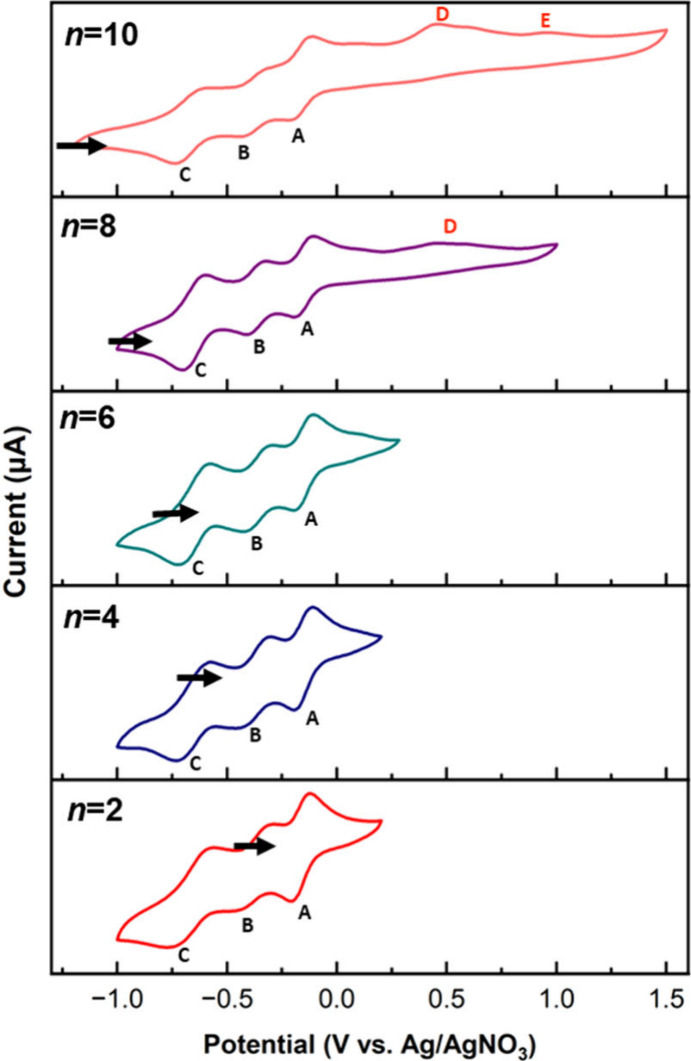
Cyclic voltammograms of **PMo**_**12**_**/*n*** for *n* = 2, 4, 6, 8, and 10 in a 100 mM solution of (TBA)PF_6_ in MeCN. [POM] = 1 mM. Working electrode (WE): glassy carbon. Reference electrode (REF): Ag/AgNO_3_. Counter electrode (CE): Pt. Scan rate = 50 mV/s. Arrows indicate rest potentials and the initial scan direction

Cyclic voltammograms for the most soluble **PMo**_**12**_**/*n*** products with *n* = 2, 4, 6, 8, and 10 are shown in Figure 8 and summarized in Table S5. Three characteristic waves (A–C) shift to more positive potentials by 10–15 mV from *n* = 2 to *n* = 6, and the remaining potentials become more negative with increasing degrees of reduction. The effect of added Li^+^ on the voltammetry of (TBA)_3_[PMo_12_O_40_] was studied by Himeno and co-workers, who observed a transition from separate one-electron to two-electron (or overlapping one-electron) waves in acetone and acetonitrile as [Li^+^] was increased from 0 to 100 mM. This was ascribed to association of Li^+^ with [PMo_2_^V^Mo^VI^_10_O_40_]^(3+*n*)–^ when *n* ≥ 2.^76^ For the solutions of reduced **PMo**_**12**_**Li**_***x***_ species in these cyclic voltammetry studies, [Li^+^] = *n*[POM], i.e., from 2 to 10 mM, so similar effects should be observed, although attempts to determine the number of electrons associated with each wave by chronopotentiometry were inconclusive due to the lack of well resolved inflections.

Strong evidence for Li–O interactions in the **PMo**_**12**_**/2** product was obtained from the X-ray crystal structure of (TBA)_4_[PMo_12_O_40_{Li(MeCN)}] obtained by recrystallization from MeCN (Figure S6, Table S6). One tetragonal face of the **α-PMo**_**12**_**(II)** anion is capped by Li^+^ with Li–O bonds of 1.99(2) Å, and the absence of Mo–Mo bonding is confirmed by the Mo–Mo distances of >3.5 Å. More extensive Li–O bonding, including to terminal Mo=O bonds, would therefore be expected for **PMo**_**12**_**Li**_***x***_ species formed at higher degrees of reduction, but we have yet to obtain crystals for single-crystal X-ray diffraction

A notable feature of the voltammograms for *n* = 8 and 10 is the appearance of irreversible oxidation peaks at potentials >400 mV versus Ag/AgNO_3_ (Figure 8, peaks D and E). The peak for **PMo**_**12**_**/8** is at 458 mV, while **PMo**_**12**_**/10** shows two peaks at 454 and 967 mV versus Ag/AgNO_3_. It has been proposed that irreversible peaks at ∼400 mV versus Ag/AgCl in the cyclic voltammograms of Keggin-type tungstates are characteristic of metal–metal bonded “heteropoly brown” species.^9,12^ The features D and E in Figure 4 therefore suggest the formation of metal–metal bonded {Mo^IV^_3_} triads in the **PMo**_**12**_**/*n*** products when *n* ≥ 8, and to our knowledge, this is the first electrochemical evidence for {Mo^IV^_3_} triads in an α-Keggin-type molybdate.

In order to determine the overall degree of reduction of the **PMo**_**12**_**/*n*** products with *n* = 2, 6, 8, and 12, potentiometric redox titrations against (NH_4_)_2_[Ce(NO_3_)_6_] were carried out for solutions in MeCN (Figure S7). Results were broadly consistent with the number of mole equivalents of added Li, implying stoichiometric transfer of electrons from Li for these reactions.

## Discussion

These investigations were undertaken to establish the feasibility of a solvent-free, incremental injection of electrons into **PMo**_**12**_ from Li metal to give reduced **PMo**_**12**_**Li**_***x***_ species. Results from XANES analysis of the solid products obtained by ball-milling **PMo**_**12**_ with *n* mole equivalents of Li metal (Figure 3b) are consistent with the efficient addition of *n* electrons and a steady decrease in the *average* oxidation state of Mo. Potentiometric redox titrations of the soluble **PMo**_**12**_**/*n*** reduction products (*n* = 2, 6, 8, and 12) support this assertion, but solid-state ^31^P NMR spectra of **PMo**_**12**_**/*n*** products indicate that electron transfer from Li during these mechanochemical reactions is not homogeneous, and these solids are mixtures. This may be due to particle size effects on the electron transfer from the Li surface during the early stages of milling coupled with slow inter-POM electron exchange kinetics in the solid state.

Detailed analysis by Penfold and co-workers of the operando XAS data from studies of a **PMo**_**12**_-based Li ion battery cathode indicated that the main structural changes expected upon the addition of up to 12 electrons to α-[PMo_12_O_40_]^3–^ are small increases in the Mo–Mo distances and Mo–O bond lengths while electrons are delocalized, but further reduction to give super-reduced species was predicted to result in significant structural rearrangement through the formation of Mo–Mo bonds.^51^ The theoretical analysis of **PMo**_**12**_**(XXIV)** gave a best fit for the formation of nine Mo–Mo bonds within three {Mo^IV^_3_} triads rather than the four suggested by Awaga and co-workers.^17,18^ Our XANES, EXAFS, and XPS data are consistent with the formation of a metal–metal-bonded {Mo^IV^_3_} triad at a lower degree of reduction than that predicted by Penfold and co-workers, but XPS results show that insufficient Mo^IV^ is present in the super-reduced **PMo**_**12**_**/*n*** products for there to be three {Mo^IV^_3_} units in the **PMo**_**12**_**Li**_***x***_ species. The Mo^V^ observed in our super-reduced samples is therefore likely to be present in Mo^V^–Mo^V^ bonds, as are observed in ε-Keggin-type structures. Bands associated with Mo^V^–Mo^V^ and {Mo^IV^_3_} in UV–vis spectra of MeCN solutions of **PMo**_**12**_**/*n*** products from *n* = 10 to *n* = 24 provide more evidence of these structural rearrangements of the POM framework. From the EXAFS spectra, the most significant structural change occurs after addition of >12 mol equiv of Li in the reduction reactions, which is broadly consistent with the theoretical analysis and coincides with changes in the solution ^31^P NMR and UV–vis spectra.

The structural changes upon reduction are evident in the IR spectra of the **PMo**_**12**_**/*n*** reduction products shown in Figure 2. Similar reductions in the intensity of ν(P–O) and ν(Mo–O–Mo) peaks were reported for spectroelectrochemical studies of (TBA)_3_**PMo**_**12**_ reduction in MeCN by up to four electrons,^50^ and for the reduction of K_3_PMo_12_O_40_ by up to ∼7 electrons per anion with H_2_ at 300–400 °C for 3–50 h.^77^ For two-electron reduction of **PMo**_**12**_, it was proposed that the gross structure is not affected by the addition of the electrons and that observed changes in the IR spectrum arise from coupling of the motion of the delocalized, diamagnetically paired electrons (a bipolaron) with the bridging vibrations in the anion framework.^10^ This may also be the case for **PMo**_**12**_**Li**_***x***_ species where *x* > 2 while the electrons remain delocalized, but distortions due to the formation of a {Mo^IV^_3_} unit within the framework should result in new ν(Mo–O–Mo) bands and reduced intensity of terminal ν(Mo=O) bands. The shifts of ν(Mo=O) and ν(Mo–O–Mo) bands to lower wavenumbers are consistent with a weakening of Mo–O bonds upon reduction of Mo^VI^, a general effect when the anionic charge on a POM is increased, which will be enhanced by Li–O interactions. The framework oxygens in reduced [PMo_12_O_40_]^(3+*n*)–^ become increasingly basic with the addition of electrons, and interactions with cations must be considered.^27^ Interactions with Li^+^ are expected to become stronger with increasing degrees of reduction and might be compared with the protonation of terminal W^VI^=O which accompanies the formation of metal–metal bonded {(W^IV^–OH_2_)_3_} triads in six-electron-reduced tungstate Keggin anions. The appearance of broad features in the IR spectra at wavenumbers ∼1100 and <600 cm^–1^ for more highly reduced **PMo**_**12**_**/12** products may be associated with the formation of LiOMo bonds, while the decrease in intensity of the ν(Mo=O) band concomitant with the lengthening of the terminal Mo=O bonds observed by EXAFS provides further evidence for the formation of Mo^IV^–Mo^IV^ bonds.^21b^

It is evident from solution ^31^P NMR spectra of the **PMo**_**12**_**/*n*** products in propylene carbonate that rapid electron redistribution results in the observation of single peaks from *n* = 2 to *n* = 12 upon dissolution. Hence, the mixture of **PMo**_**12**_, **PMo**_**12**_**(I)**, and **PMo**_**12**_**(II)** in the solid **PMo**_**12**_**/1** product, with an *average* degree of reduction of one electron, is cleanly converted to only **PMo**_**12**_**(I)**. For related 1- and 2-electron reduced tungstate systems, Kozik and Baker showed from ^31^P NMR line-width analysis that aqueous exchange rates for **PW**_**12**_/**PW**_**12**_**(I)** and **PW**_**12**_**(I)**/**PW**_**12**_**(II)** increase with ionic strength.^78^ The chemical shift for the **PMo**_**12**_**/4** product in propylene carbonate (−6.66 ppm) suggests an α-Keggin structure, whereas the stable **β-PMo**_**12**_**(IV)** isomer obtained by reduction of H_3_PMo_12_O_40_ by four electrons in water or water/dioxane has a chemical shift of approximately −13 ppm.^29,79^ At higher degrees of reduction (*n* > 12) the significant switch to positive chemical shifts and the much broader lines (Figure 6) are suggestive of a significant structural/chemical change, which aligns with EXAFS results. The broad peaks could be due to long correlation times caused by aggregation of reduced anions through bridging Li–O bonding or to the magnetic properties of the super-reduced species. However, while magnetic suspectibility measurements on **PMo**_**12**_**/*n*** products in MeCN using the Evans NMR method (Table S7) are consistent with the expected paramagnetic **PMo**_**12**_**(I)** for *n* = 1 and diamagnetic **PMo**_**12**_**(II)** for *n* = 2, data for *n* = 4, 6, 8, and 20 show less paramagnetism than for **PMo**_**12**_**(I)**, indicative of spin-paired **PMo**_**12**_**Li**_***x***_ species with traces of paramagnetic impurities. This suggests that the line broadening in the ^31^P NMR spectra of **PMo**_**12**_**/20** shown in Figure 6 and Figure S5 is not simply a paramagnetic effect.

There are significant differences between the features of **PMo**_**12**_**/*n*** products observed in these studies and those proposed by Awaga and co-workers on the basis of operando XAS studies of the (TBA)_3_[PMo_12_O_40_]/carbon black/poly(vinylidene fluoride) cathode in a “molecular cluster battery” and associated DFT and molecular dynamics calculations.^17,18^ Our results indicate that not all terminal Mo=O bonds are lost, and only one or two {Mo^IV^_3_} triads are formed, whereas in the battery environment loss of all terminal Mo=O and formation of four {Mo^IV^_3_} triads was proposed. We suggest that higher degrees of reduction with lithium metal cause structural rearrangement of the {PMo_12_O_40_} framework to give ε-Keggin-type structures. It is worth noting in this regard that the previous DFT results highlighted a minimum energy structure containing two {Mo^IV^_3_} triads, two individual Mo–Mo single bonds and one Mo=Mo double bond.^18^

Under solvent-free conditions, the extensive Li–O interactions required to transform terminal Mo=O to Mo–O single bonds are likely ultimately to produce basic metastable lithium oxide phases that could promote decomposition of the TBA cations, e.g., by Hoffman elimination to give butene and tributylamine. When (TBA)_3_[PMo_12_O_40_] was ball-milled in the absence of Li, the FTIR, UV–vis, and ^31^P NMR spectra showed no signs of decomposition (Figure S8). XPS data for **PMo**_**12**_**/*n*** products with *n* = 16–24 indicate that the Mo(II) detected (Figure 5) is due to generation of Mo_2_C as a separate phase, rather than as an organometallic group formed within the super-reduced POM. The generation of Mo_2_C and the relatively constant atomic percentages of Mo^VI^, Mo^V^, and Mo^IV^ in these samples suggest that reactive Mo sites generated in super-reduced species might be reacting with products from TBA decomposition. It is interesting to note that reduction of silica-supported MoO_3_, often prepared from soluble molybdates, can generate active Mo^IV^, which reacts with alkenes to give surface alkylidene species.^80^

## Conclusions

In this work, we have successfully demonstrated stepwise solid-state mechanochemical reduction of (TBA)_3_[PMo_12_O_40_] with lithium metal for the first time. The mixtures of reduced species observed in the **PMo**_**12**_**/*n*** solid products give simpler solution ^31^P NMR spectra, indicative of slow electron exchange during the solid reaction mixtures but rapid electron redistribution upon dissolution. XAS and XPS studies confirmed initial reduction from Mo^VI^ to Mo^V^ and further reduction to Mo^IV^ when *n* ≥ 8–10. EXAFS, UV–visible spectroscopy, and cyclic voltammetry provided evidence for the formation of Mo^V^–Mo^V^ and Mo^IV^–Mo^IV^ bonds from *n* ∼ 8, but XPS data indicated that at *n* = 24 only one or possibly two {Mo^IV^_3_} triads are formed within the structure alongside Mo^V^–Mo^V^ bonds. This is consistent with the loss of some but not all of the terminal M=O bonds as observed by IR and EXAFS spectroscopy. The combined results from these studies are consistent with rearrangement of the POM framework to an ε-Keggin-type structure upon super-reduction and reveal similarities to electron-rich systems obtained from trinuclear {Mo^IV^_3_} metal–metal bonded complexes, which do not contain central phosphorus.^21b^

The presence of Mo_2_C in the **PMo**_**12**_**/*n*** products for *n* = 16–24 was somewhat surprising. We propose that this is due to two factors: (i) elevated basicity at high [Li^+^] causing TBA degradation and (ii) reactions between TBA degradation products and reduced Mo sites resulting in dissociation of the Keggin framework. This could explain the apparent behavior of **PMo**_**12**_ as an electron buffer, in which the amounts of Mo^V^ and Mo^IV^ become almost constant at higher degrees of reduction as the extra electrons injected generate active Mo sites which react to form Mo–C bonds.

Mechanochemical access to electron-rich polyoxometalates will facilitate explorations of super-reduced POM chemistry, and preliminary investigations indicate that the **PMo**_**12**_**/*n*** products are readily converted to capped derivatives [PMo_12_O_40_{ML_*x*_}_*y*_]^z–^.^27^ Work is ongoing to fully characterize the super-reduced products and understand TBA cation stability under these conditions, which is crucial for product control in these mechanochemical reactions.

## Supporting Information Available

General experimental methods, XPS, EXAFS, CV, potentiometric redox titrations, solid state ^31^P MAS NMR, and crystallographic data (PDF)

## Abbreviations

ATR-FTIR: attenuated total reflectance Fourier transform infrared infrared
CV: cyclic voltammetry
NMR: nuclear magnetic resonance
XANES: X-ray absorption near edge structure
EXAFS: extended X-ray absorption fine structure
XPS: X-ray photoelectron spectroscopy
