# Supplementary material for: Mechanochemical Polyoxometalate Super-Reduction with Lithium Metal
Source: J Am Chem Soc. 2024 Sep 10;146(38):26485–96. doi: 10.1021/jacs.4c09998 (PMC11440509; doi:10.1021/jacs.4c09998)
Supplement: Supplementary file 1 — ja4c09998_si_001.pdf [file ja4c09998_si_001.pdf]

SUPPORTING INFORMATION

Mechanochemical Polyoxometalate Super-Reduction with Lithium Metal

Magda Pascual-Borràs,[1] Elisabetta Arca[2], Hirofumi Yoshikawa[3], Thomas Penfold[1,] Paul G. Waddell[1] and R. John Errington[1*]

[1]NUPOM Lab, Chemistry, School of Natural & Environmental Sciences, Newcastle University, NE1 7RU, Newcastle Upon Tyne, UK

[2]School of Mathematics, Statistics and Physics, Newcastle University, NE1 7RU, Newcastle Upon Tyne, UK

[3]Department of Materials Science, Kwansei Gakuin University, Sanda, Hyogo 669-1330, Japan

Contents

page

Experimental 2-4

Tables

Table S1. Crystal data and structure refinement for $(PPN)_3[PMo_{12}O_{40}]$ 5

Table S2. Bond lengths associated with $\{Mo^{VI}_3\}$ triads within the structure of $(PPN)_3[PMo_{12}O_{40}]$ 6

Table S3. Interatomic distances (Å) for bands in EXAFS spectra of **PMo_{12}/*n*** products 7

Table S4. Summary of the average of Δ_{BE} for molybdenum oxides 18

Table S5. Reduction potentials (V) for **PMo_{12}/*n*** products vs. $Ag/AgNO_3$ 18

Table S6. Crystal data and structure refinement for $(TBA)_4[PMo_{12}O_{40}\{Li(NCMe)\}]$ 19

Table S7. Magnetic Susceptibilities for **PMo_{12}/n** products at 25 °C. 20

Figures

Figure S1. The asymmetric unit of the structure of $(PPN)_3[PMo_{12}O_{40}]\cdot MeCN\cdot 0.65\ H_2O$ 5

Figure S2. Mo K-edge EXAFS functions of $^3\chi(\kappa)$ functions for **PMo_{12}/*n*** products 6

Figure S3. Wavelet transform of κ^3-weighted EXAFS spectra for **PMo_{12}/*n*** products 8

Figure S4. XPS spectra for **PMo_{12}/n** products (a) Mo3d (b) O1s (c) P2p (d) C1s (e) Li1s 9-17

Figure S5. Solid-State ^{31}P NMR of the soluble and insoluble components obtained from PMo_{12}/n products (*n* = 12 – 24) after extraction into acetonitrile, filtration and evaporation of the filtrate 18

Figure S6. The anion in the structure of $(TBA)_4[PMo_{12}O_{40}\{Li(NCMe)\}]$ with ellipsoids drawn at the 50% probability level 19

Figure S7. Titrations of **PMo_{12}/*n*** products in MeCN with 0.01 *M* CAN in MeCN 20

Figure S8. (a) ATR FTIR spectra and (b) UV-vis spectra of $(TBA)_3[PMo_{12}O_{40}]$ before and after ball-milling, and (c) ^{31}P NMR spectrum of ball-milled $(TBA)_3[PMo_{12}O_{40}]$ 21

References 22

SUPPORTING INFORMATION

General

Reactions and manipulations were carried out under dry, oxygen-free argon using standard Schlenk and dry-box techniques unless stated otherwise.[1] Acetonitrile was dried and distilled from calcium hydride and then stored over 3A molecular sieves. Propylene carbonate was purged with argon then stored over 3A molecular sieves for at least three days prior to use. Lithium metal in paraffin (> 99%) was purchased from Scientific Laboratory Supplies and handled under argon in an MBraun glove box. The metal surface was scraped clean and cut into small (1 - 3 mm) pieces before weighing.

$(TBA)_3[PMo_{12}O_{40}]$ and $(PPN)_3[PMo_{12}O_{40}]$ were prepared following a literature procedure using either (TBA)Br or (PPN)Cl to precipitate the products, which were recrystallised from MeCN.[2]

Mechanochemical reactions

Caution: reactions between electropositive elements and metal oxides may result in highly exothermic, self-propagating reactions (as in thermite reactions) and should be tried on a small scale initially to establish safe procedures before scale-up.

$(TBA)_3[PMo_{12}O_{40}]$ was mixed in an argon-filled MBraun glovebox with from 1 to 24 mole-equivalents of lithium metal and sealed into a stainless steel 25 mL screw-top reaction jar with two 10 mm stainless steel balls before ball-milling in a Retsch MM400 mixer mill at 25-30 Hz oscillation frequency for 60 min at room temperature. To avoid excessive exotherms, the reactions with ≥ 16 equivalents of lithium were performed stepwise by initially milling $(TBA)_3[PMo_{12}O_{40}]$ with 12 equivalents of lithium for 60 min., opening the jar in the glovebox and adding the required extra amount of lithium before milling for a further 60 min.
Solid products from mechanochemical reactions were removed from the milling jar in the glovebox containing the potentiostat and FTIR and UV-visible spectrometers.

Characterisation

Infrared spectroscopy

FTIR spectra were recorded from powders on a Bruker Alpha FTIR spectrometer fitted with a Platinum ATR attachment.

Solid State and Solution Nuclear Magnetic Resonance (NMR)

^{31}P NMR spectra of solutions were recorded on Bruker Avance III 300 MHz or Bruker Avance III HD 500 MHz NMR spectrometers operating at 121.49 and 202.46 MHz, respectively. Samples were referenced to 85% H_3PO_4. A relaxation delay of 30 s was used and the number of scans was 32 (at 121.49 MHz) or 1024 (at 202.46 MHz).
Solid-state ^{31}P MAS NMR spectra were recorded at 202.40 MHz using a Bruker Avance III HD 500 NMR spectrometer with 4 mm rotors. They were obtained using cross-polarisation (CP) and single pulse with a 5 s (CP) and 60 s (single pulse) relaxation delay, 3.4-5.0 ms contact time at ambient probe temperature (~25 °C) and at a MAS rate of 5-10 kHz. Spectra were referenced to an external sample of neat tetramethylsilane (carried out by setting the high-frequency signal from adamantane to 37.8 ppm).
Less soluble **PMo₁₂/n** products (n = 12 - 24) were extracted into acetonitrile, filtered, and the solution was pumped dry. Solid-state ^{31}P NMR spectra of both fractions were recorded.

UV-VIS spectroscopy

UV-VIS spectra of 0.2 m*M* solutions of **PMo_{12}/n** products were recorded in 1 cm cuvettes using an Ocean-HDX-XR UV to NIR spectrometer and DH2000 light source.

Potentiometric Titrations

The degree of reduction of **PMo_{12}/*n*** products was determined by potentiometric redox titration against $(NH_4)_2[Ce(NO_3)_6]$ (CAN) in MeCN. Samples of 0.2 - 1 m*M* **PMo_{12}/*n*** in acetonitrile were titrated with a 0.01 *M* CAN in acetonitrile in a mini (3 mL) cell using a glassy carbon electrode and an $Ag/AgNO_3$ reference electrode. For incompletely soluble products when n > 12, solid suspensions were obtained and the potentiometric titrations were unreliable.

SUPPORTING INFORMATION

Cyclic Voltammetry

Cyclic Voltammetry was performed with a PalmSens4 potentiostat using 100 m*M* (TBA)[BF_4] in dry acetonitrile as electrolyte. Measurements were performed at room temperature in a conventional three-electrode cell in an argon glove box. A glassy carbon (GC) electrode with a diameter of 3mm was used as a working electrode with a Pt wire auxiliary electrode. Potentials are quoted against a Ag/$AgNO_3$ reference electrode. All analytes were prepared as 1 m*M* solutions in the electrolyte solution.

X-ray Photoelectron Spectroscopy

X-ray photoelectron spectroscopy (XPS) measurements were performed using a Thermo Scientific XPS instrument operated at a base pressure better than 5×10^{-9} Torr using an Al *K*α source (λ =1486.6 eV). Due to the limited conductivity of the powder samples, a charge neutraliser was used, which brings up the pressure during measurements to 3×10^{-7} Torr. All samples were loaded in an air-tight sample holder inside an argon glovebox and immediatey transferred into the XPS system via an air-free transfer mechanism. All presented data were acquired using a pass energy of 40eV and 0.1eV step size. We investigated the sensitivity of the sample toward X-ray damage and the potential gain in resolution by acquaring two sets of scans at lower resolution (40eV pass energy) and higher resolution (20eV pass energy), using a longer integration time for higher resolution scans. By comparing the amount of Mo(V) present in both sets of data, we concluded that the initial beam damage plateaus during the acquisition time for the first set of data (pass energy 40eV), with little or no variation in the amount of Mo(V) visible in the set acquired at 20 eV pass energy. We also noticed that we could attribute all phases with confindence using 40eV pass energy, with no substaintial gain by using 20eV pass energy. Therefore we decided to conduct all measurements using 40eV pass energy, to keep the X-ray exposure to the minimum.
Curve fitting was performed using custom-written procedures in the Wavementrics IgorPro software package.[3] For the Mo 3d and P 2p, the characteristic spin-orbit components were included in the fitting routing, using a binding energy separation between the Mo $3d_{5/2}$ and Mo $3d_{3/2}$ components of 3.15 eV and a separation 0.86 eV for P $2p_{3/2}$ and $2p_{1/2}$ components. The ratio between the two spin orbital components set to 3 to 2 for Mo and 2 to 1 for P. Phase assignment was based on the characteristic binding energy separation that chemical species present.

X-ray Absorption Spectroscopy

XAFS measurements for the Mo *K*-edge were performed in transmission mode at room temperature using the beamline BL14B1 of the SPring-8 at an electron energy of 8.0 GeV and an ring current of 100 mA. A Si (311) fixed-exit double-crystal monochromator was used to select a specific X-ray energy. (or Incident beam energies from 19708 to 20973 eV were selected by a Si (311) double-crystal monochromator) The intensities of incident(I_0) and transmitted(I_t) X-rays were measured using ionization chambers filled with a Ar/N_2 mixture (1:1). The X-ray spot size was reduced to 1.6 mm × 2.6 mm by a slit. Samples diluted with boron nitride and compressed to form a 10 mm diameter pellet were placed between two transmission ion chambers. One Mo *K*-edge spectrum was obtained within 546 sec. Mo metal, MoO_2, MoO_3 and $TBA_3[PMo_{12}O_{40}]$, diluted with boron nitride were used as reference materials to calibrate the relationship between the absorption energy of Mo *K*-edge and the averaged oxidation states of Mo.

X-ray absorption near edge structure (XANES) spectra were obtained by pre-edge background subtraction and subsequent normalization using the software Athena. Extended X-ray absorption fine structure (EXAFS) spectra were obtained by the standard procedures using Ifeffit (Artemis) program. The k^3-weighted Mo *K*-edge EXAFS oscillation was obtained by pre-edge baseline subtraction, edge-energy determination, post-edge background subtraction, and normalization with the atomic absorption coefficients. The extracted EXAFS

oscillation (2-14 Å^{-1}) were Fourier transformed into R-space. Curve-fitting analysis was carried out by using the backscattering amplitudes and the phase shifts for Mo−O and Mo−Mo shells, which were calculated from the crystal structure of $TBA_3[PMo_{12}O_{40}]$.
The Wavelet analysis was performed using an in-house code.[4] A Morlet wavelet with the parameters η=10.5 and σ =1.5 was used throughout.

Single Crystal X-Ray Diffraction

Single crystal diffraction data for $(PPN)_3[PMo_{12}O_{40}]\cdot MeCN\cdot 0.65H_2O$ and $(TBA)_4[PMo_{12}O_{40}\{Li(NCMe)\}]$ were collected on an Xcalibur, Atlas, Gemini ultra diffractometer at 150 K using an Oxford Cryosystems CryostreamPlus open-flow N_2 cooling device using molybdenum radiation ($\lambda_{MoK\alpha}$ = 0.71073 Å) and copper radiation ($\lambda_{CuK\alpha}$ = 1.54184 Å) respectively.
Intensities were corrected for absorption using a multifaceted crystal model created by indexing the faces of the crystal for which data were collected.[5] Cell refinement, data collection and data reduction were undertaken via the software CrysAlisPro.[6]
All structures were solved using XT[7] and refined by XL[8] using the Olex2 interface[9]. All non-hydrogen atoms were refined anisotropically and hydrogen atoms were positioned with idealised geometry. The displacement parameters of the hydrogen atoms were constrained using a riding model with $U_{(H)}$ set to be an appropriate multiple of the U_{eq} value of the parent atom.

Magnetic susceptibility measurements in solution

All determinations of magnetic susceptibilities in solution at room temperature were made by a modified Evan's method.[10] This method uses the parent solution of the oxidised species as a reference solution and yields the difference between the susceptibilities of the reduced and oxidised forms via the following equation:
X_M(reduced)-X_M(oxidised)= $(3\Delta_v/4\pi v_0 C) \times 10^6$
where Δ_v is the difference in frequencies of ^{t}BuOH methyl protons in the two solutions, v_0 is the radiofrequency of ^{1}H nucleus and C is the concentration in millimoles per liter.
The magnetic moment (μ_{exp}) can be obtained by the following equation:[11]

$$\mu_{exp} = \sqrt{\frac{3k}{N\beta^2}}(X_M T)$$

where χ_M is the molar magnetic susceptibility (mL/mol), *T* is temperature (K), *k* is Boltzmann constant, *N* is Avogadro's number, and β is Bohr magneton of the electron.

For each sample, a 5 - 6 m*M* solution with 2% of ^{t}BuOH was prepared. The $TBA_3[PMo_{12}O_{40}]$ solution was transferred into an NMR insert for a 5mm tube, and the solutions of $\mathbf{PMo_{12}}$/***n*** were transferred into the 5mm screw-cap NMR sample tube.

SUPPORTING INFORMATION

X-ray crystallographic data for $(PPN)_3[PMo_{12}O_{40}]$

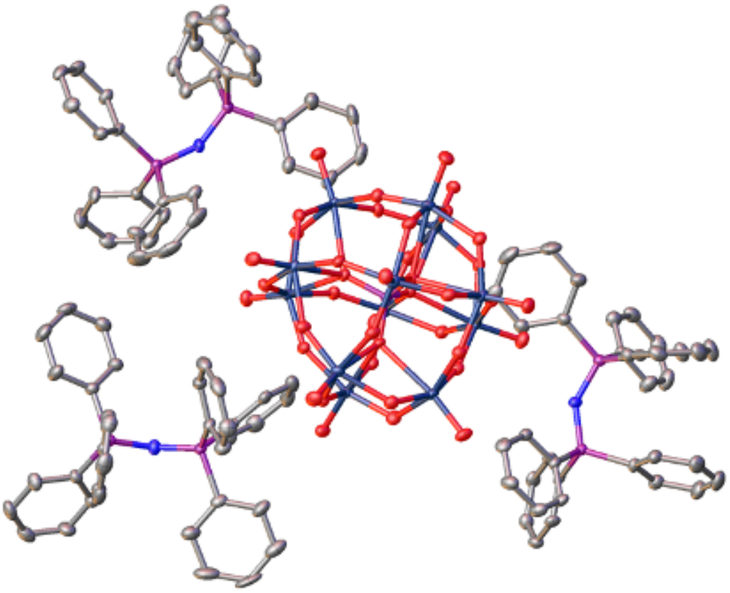

Figure S1. The asymmetric unit of the structure of $(PPN)_3[PMo_{12}O_{40}]\cdot MeCN\cdot 0.65\ H_2O$ with ellipsoids drawn at the 50% probability level. Hydrogen atoms and solvent molecules have been omitted for clarity.

Table S1. Crystal data and structure refinement for $(PPN)_3[PMo_{12}O_{40}]$

Empirical formula	$C_{110}H_{93}N_4O_{40.65}P_7Mo_{12}$
Formula weight	3489.35
Temperature/K	150.0(2)
Crystal system	Triclinic
Space group	P-1
a/Å	17.2957(4)
b/Å	18.6014(4)
c/Å	18.9808(5)
α/°	83.0064(19)
β/°	81.8520(19)
γ/°	86.6838(18)
Volume/Å^3	5994.8(2)
Z	2
ρ_{calc}g/cm^3	1.933
μ/mm^{-1}	1.388
F(000)	3430.0
Crystal size/mm^3	0.37 × 0.30 × 0.11
Radiation	Mo Kα (λ = 0.71073)
2Θ range for data collection/°	5.61 to 57.596°
Index ranges	-21 ≤ h ≤ 20, -22 ≤ k ≤ 25, -25 ≤ l ≤ 24
Reflections collected	96768
Independent reflections	26932 [R_{int} = 0.0487]
Data/restraints/parameters	26932/0/1568
Goodness-of-fit on F^2	1.075
Final R indexes [I>=2σ (I)]	R_1 = 0.0469, wR_2 = 0.0974
Final R indexes [all data]	R_1 = 0.0722, wR_2 = 0.1120
Largest diff. peak/hole / e Å^{-3}	1.95/-1.45

Table S2. Bond lengths associated with {Mo^{VI}_3} triads within the structure of $(PPN)_3[PMo_{12}O_{40}]$

	Mo–O/Å				Mo–Mo/Å	
	Mo=O	Mo–O_{int}	Mo–O_{ext}	Mo–O_P	Mo–Mo_{int}	Mo–Mo_{ext}
Mo_1	1.672	1.841, 2.011	1.825, 1.993	2.440	3.427	3.692, 3.696,
Mo_2	1.682	1.839, 1.978	1.847, 1.990	2.434	3.431	3.704, 3.714
Mo_3	1.674	1.854, 1.993	1.828, 1.986	2.447	3.419	3.709, 3.693
Mo_4	1.674	1.837, 1.998	1.817, 2.005	2.429	3.416	3.709, 3.692,
Mo_5	1.679	1.855, 2.006	1.825, 1.973	2.428	3.417	3.693, 3.704
Mo_{10}	1.677	1.844, 1.993	1.827, 2.011	2.424	3.419	3.706, 3.710
Mo_6	1.675	1.827, 2.446	1.997, 2.004	2.446	3.426	3.714, 3.693,
Mo_7	1.679	1.840, 1.999	1.821, 2.000	2.442	3.425	3.708, 3.709
Mo_{11}	1.674	1.854, 1.999	1.829, 1.982	2.423	3.411	3.706, 3.695
Mo_8	1.679	1.838, 2.019	1.822, 2.002	2.443	3.420	3.693, 3.708,
Mo_9	1.677	1.855, 1.995	1.827, 1.989	2.429	3.434	3.696, 3.709
Mo_{12}	1.681	1.844, 1.989	1.825, 1.992	2.431	3.409	3.710, 3.695

EXAFS data for PMo_{12}/n products.

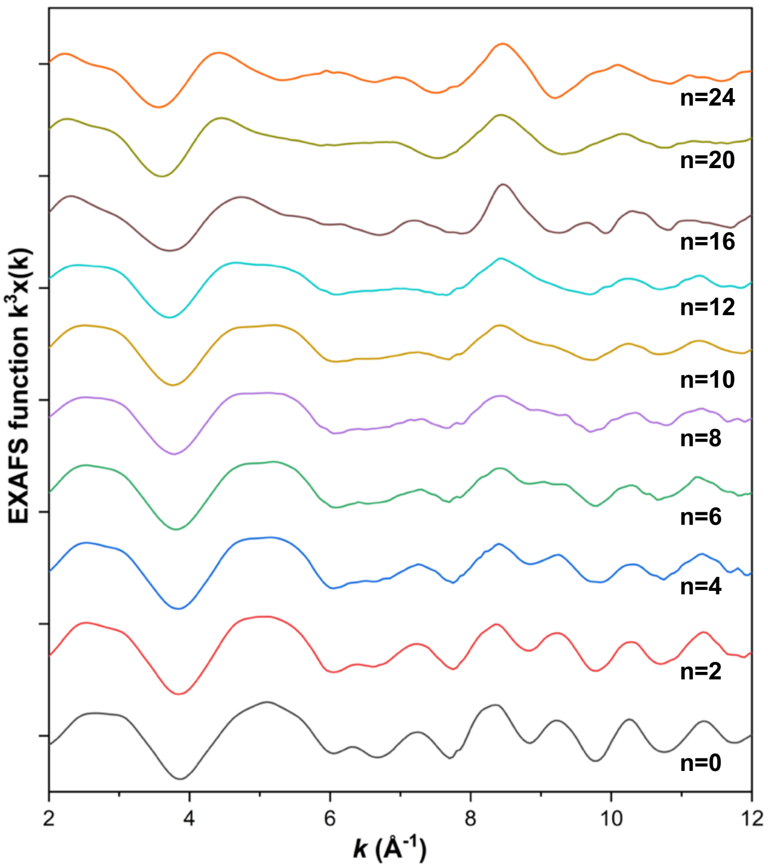

Figure S2. Mo K-edge EXAFS functions of $^3\chi(\kappa)$ functions for PMo_{12}/n products

Table S3. Interatomic distances (Å) for bands in EXAFS spectra of **PMo_{12}/*n*** products

Compound	Band A	Band B	Band C	Band D	Band E	Band F
PMo_{12}	1.03	1.51	1.93	2.30	2.70	3.21
Li_2PMo_{12}	1.04	1.47	1.77	2.36	2.70	3.13
Li_4PMo_{12}	1.04	1.47	1.74	2.33	2.70	3.09
Li_6PMo_{12}	1.04	1.44	1.74	2.33	2.70	3.09
Li_8PMo_{12}	1.01	1.44	1.68	2.33	2.70	3.09
$Li_{10}PMo_{12}$	1.01	-	1.71	2.30	2.70	-
$Li_{12}PMo_{12}$	0.98	-	1.68	2.30	2.70	-
$Li_{16}PMo_{12}$	0.98	-	1.69	2.36	-	-
$Li_{20}PMo_{12}$	0.98	-	1.66	2.27	-	-
$Li_{24}PMo_{12}$	0.95	-	1.66	2.27	-	-

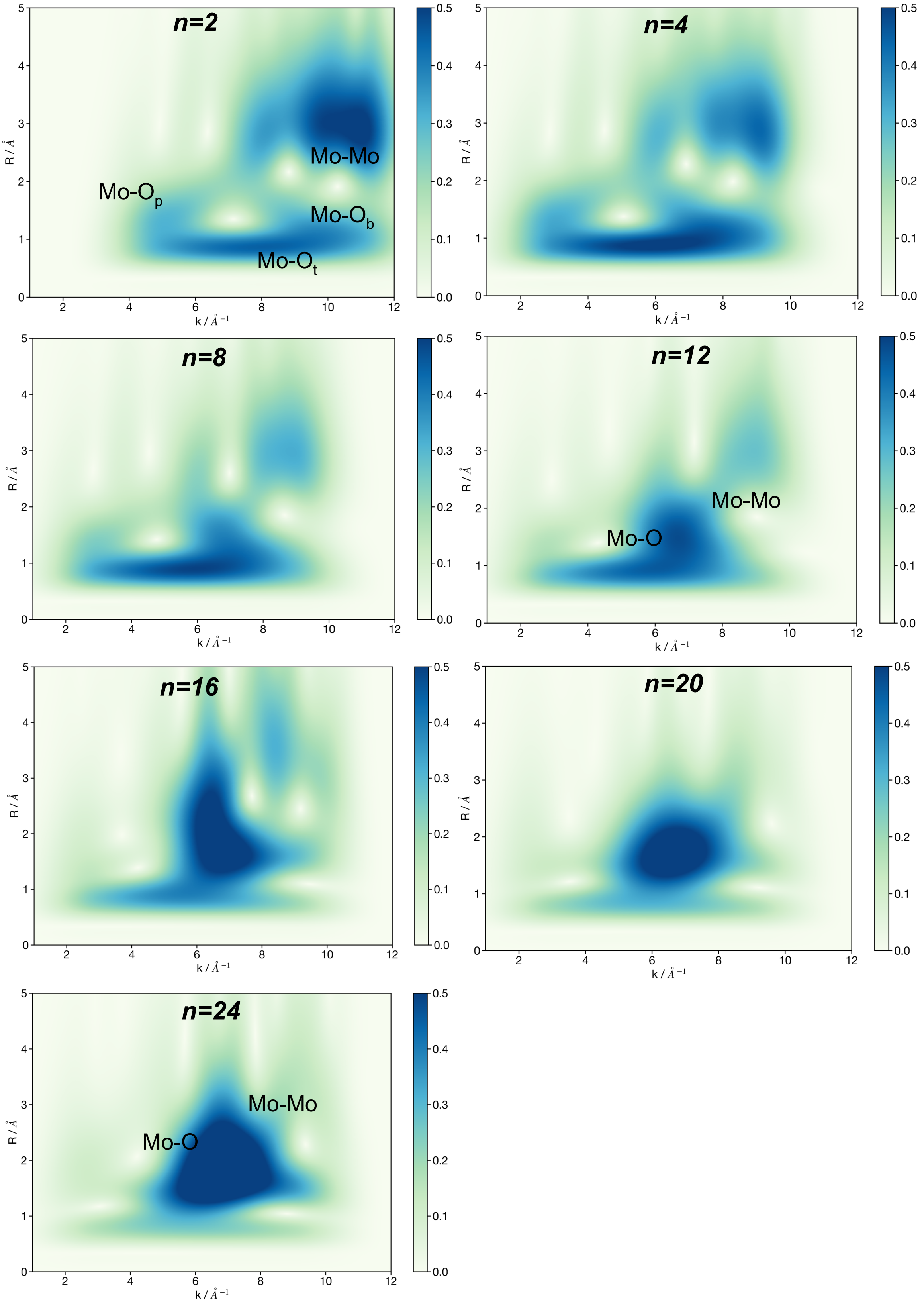

Figure S3. Wavelet transform of κ^3-weighted EXAFS spectra for **PMo_{12}/*n*** products. Analysis using Morlet wavelet and parameters η=10.5 and σ =1.5 was based upon a previously described method.[4]

SUPPORTING INFORMATION

XPS data for PMo_{12}/*n* products

a) Mo3d

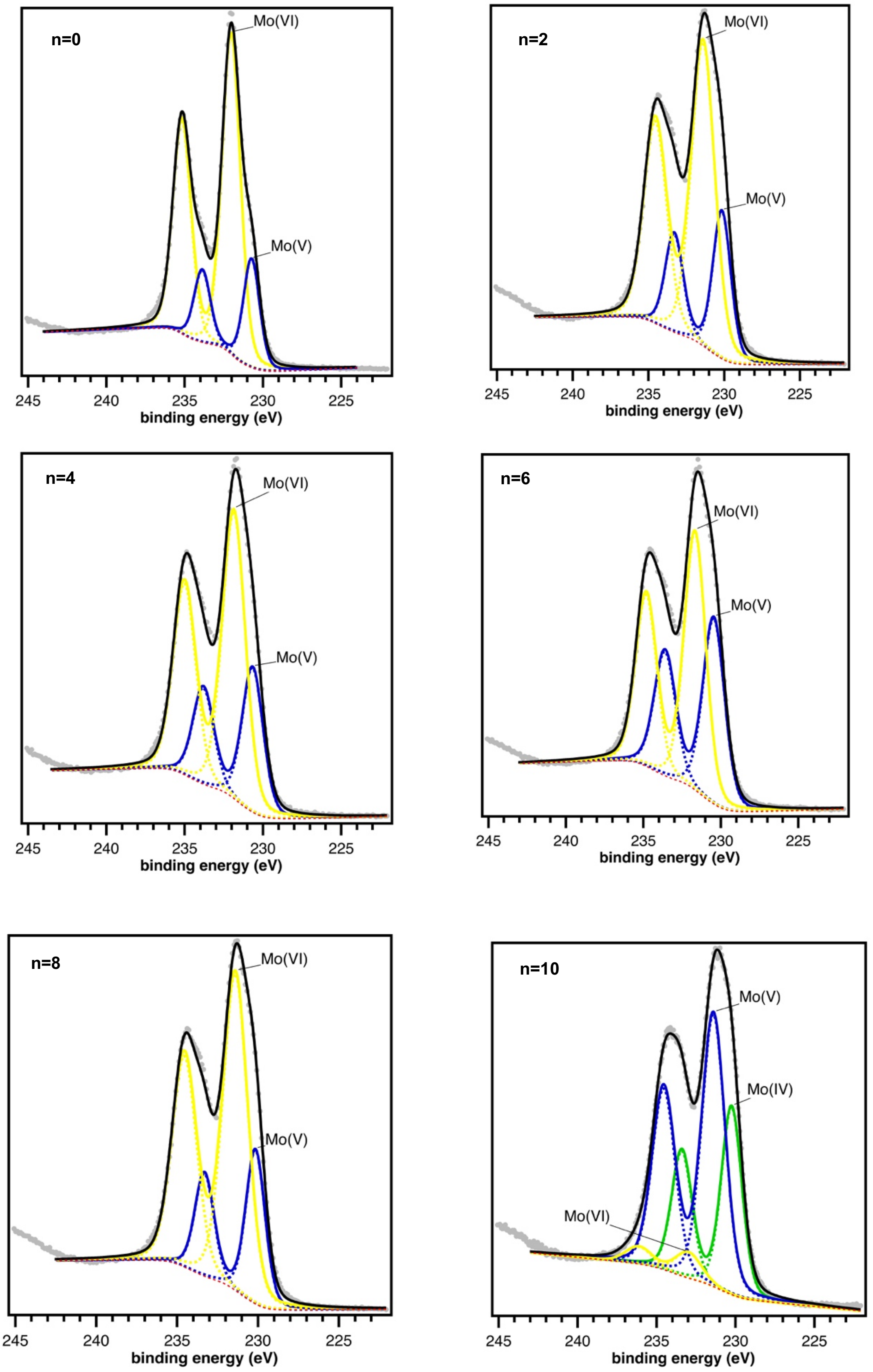

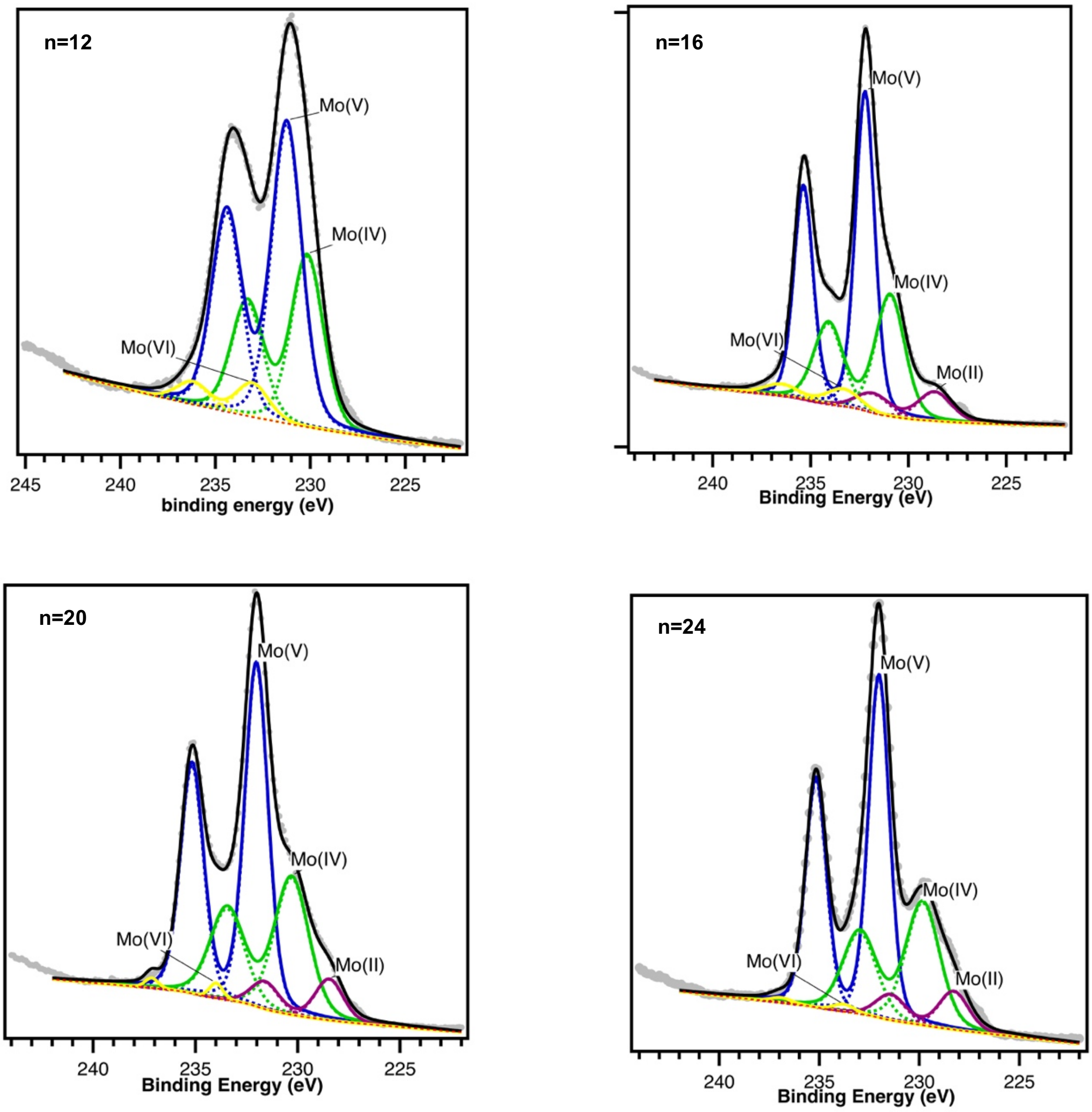
n=12
Mo(V)
Mo(IV)
Mo(VI)
245 240 235 230 225
binding energy (eV)
n=16
Mo(V)
Mo(IV)
Mo(VI)
Mo(II)
240 235 230 225
Binding Energy (eV)
n=20
Mo(V)
Mo(IV)
Mo(VI)
Mo(II)
240 235 230 225
Binding Energy (eV)
n=24
Mo(V)
Mo(IV)
Mo(VI)
Mo(II)
240 235 230 225
Binding Energy (eV)

b) O1s

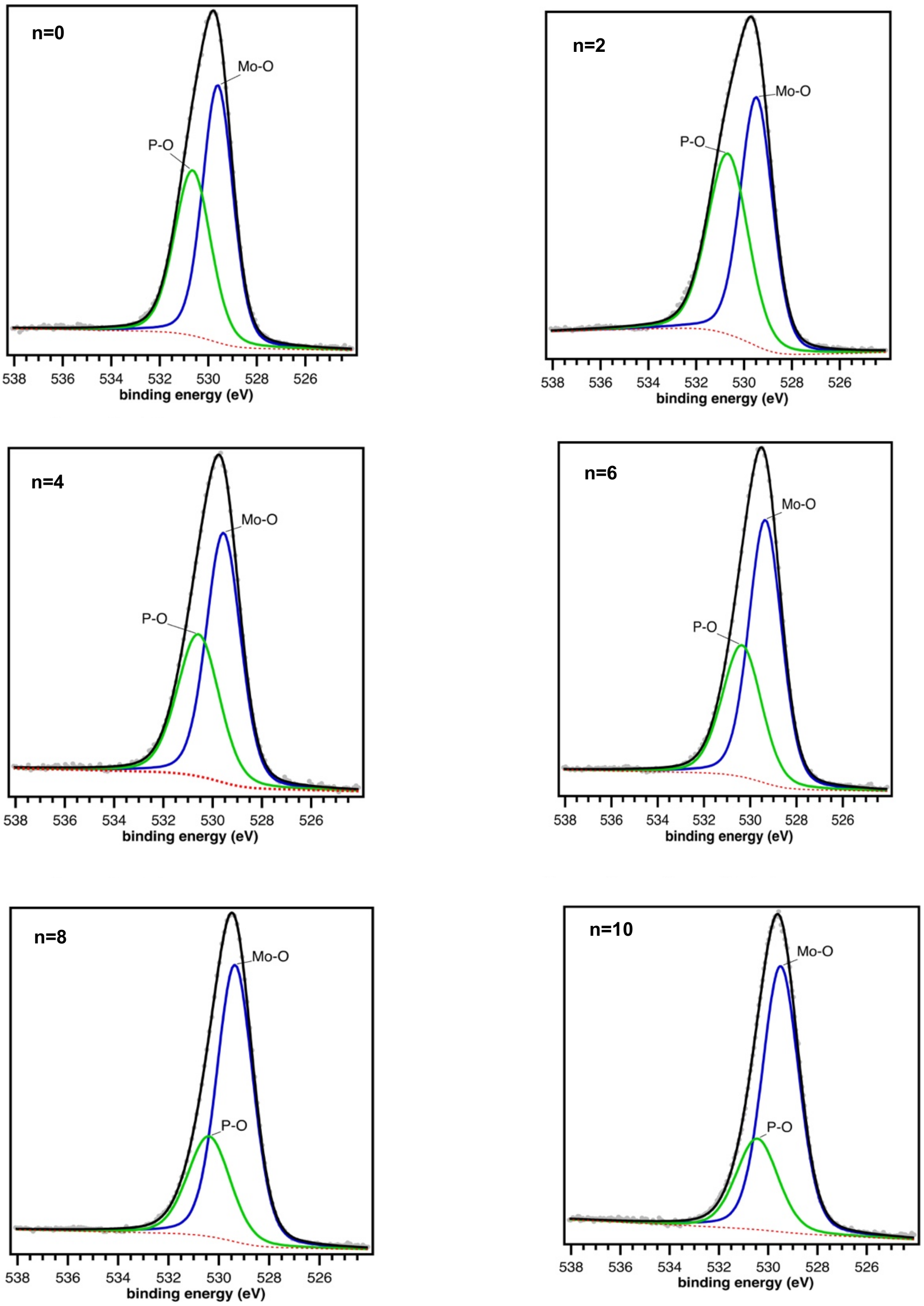
n=0
n=2
n=4
n=6
n=8
n=10
Mo-O
P-O
538 536 534 532 530 528 526
binding energy (eV)

SUPPORTING INFORMATION

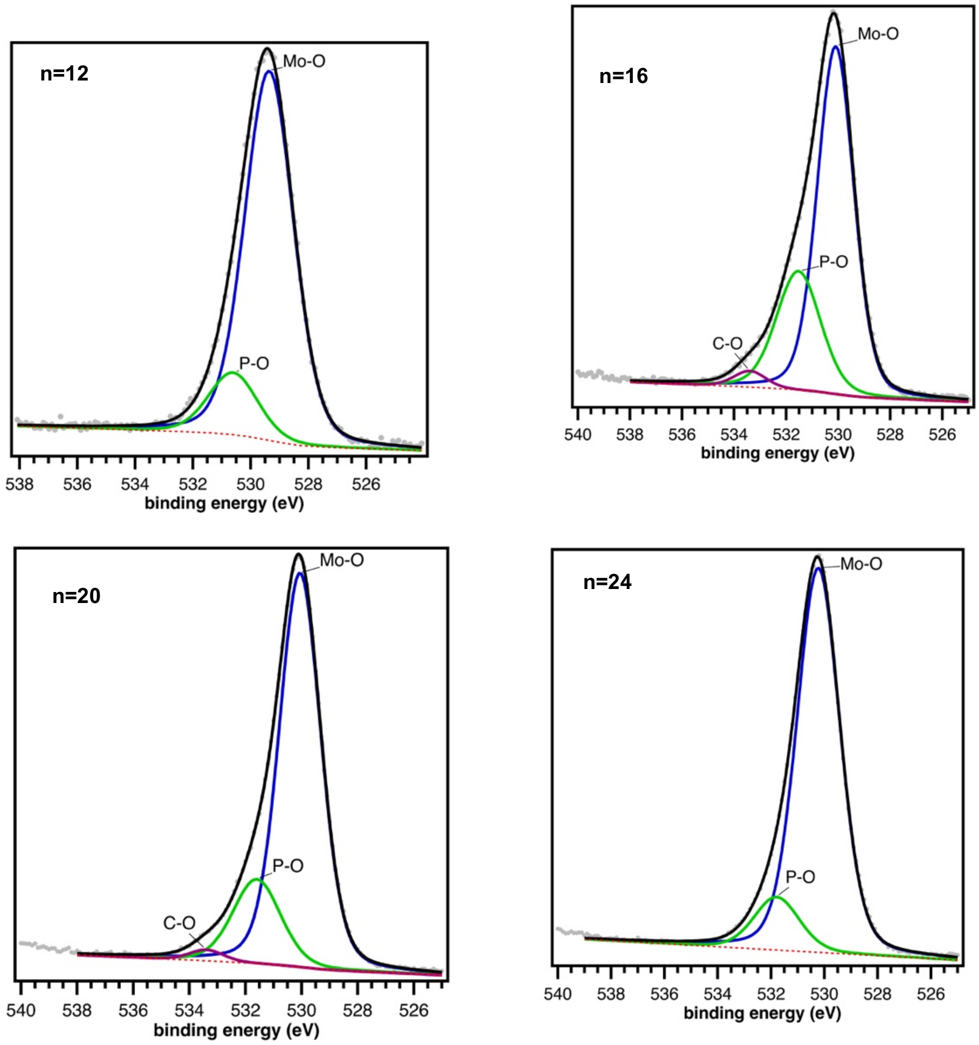

c) P2p

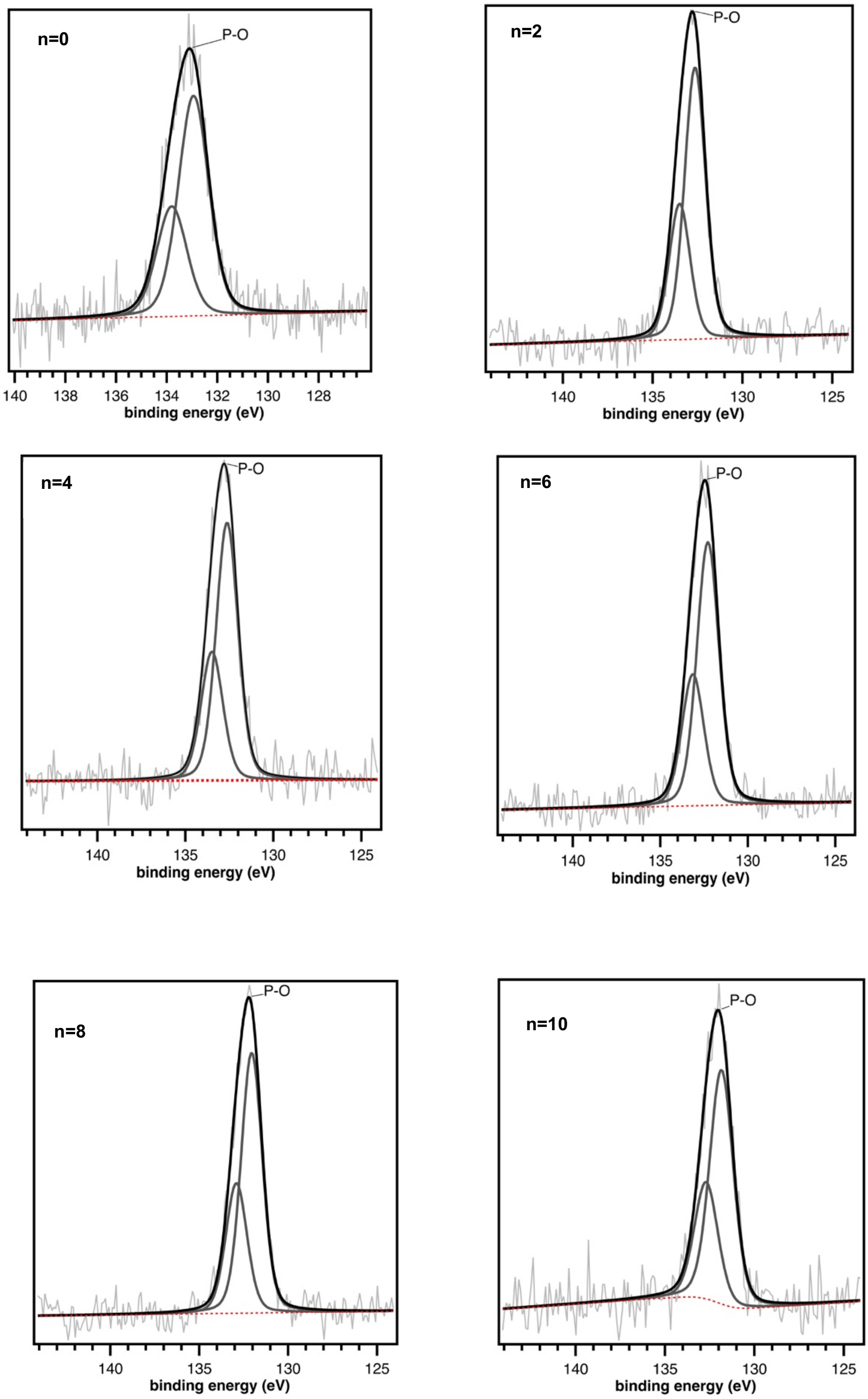
n=0
P-O
140 138 136 134 132 130 128
binding energy (eV)
n=2
P-O
140 135 130 125
binding energy (eV)
n=4
P-O
140 135 130 125
binding energy (eV)
n=6
P-O
140 135 130 125
binding energy (eV)
n=8
P-O
140 135 130 125
binding energy (eV)
n=10
P-O
140 135 130 125
binding energy (eV)

SUPPORTING INFORMATION

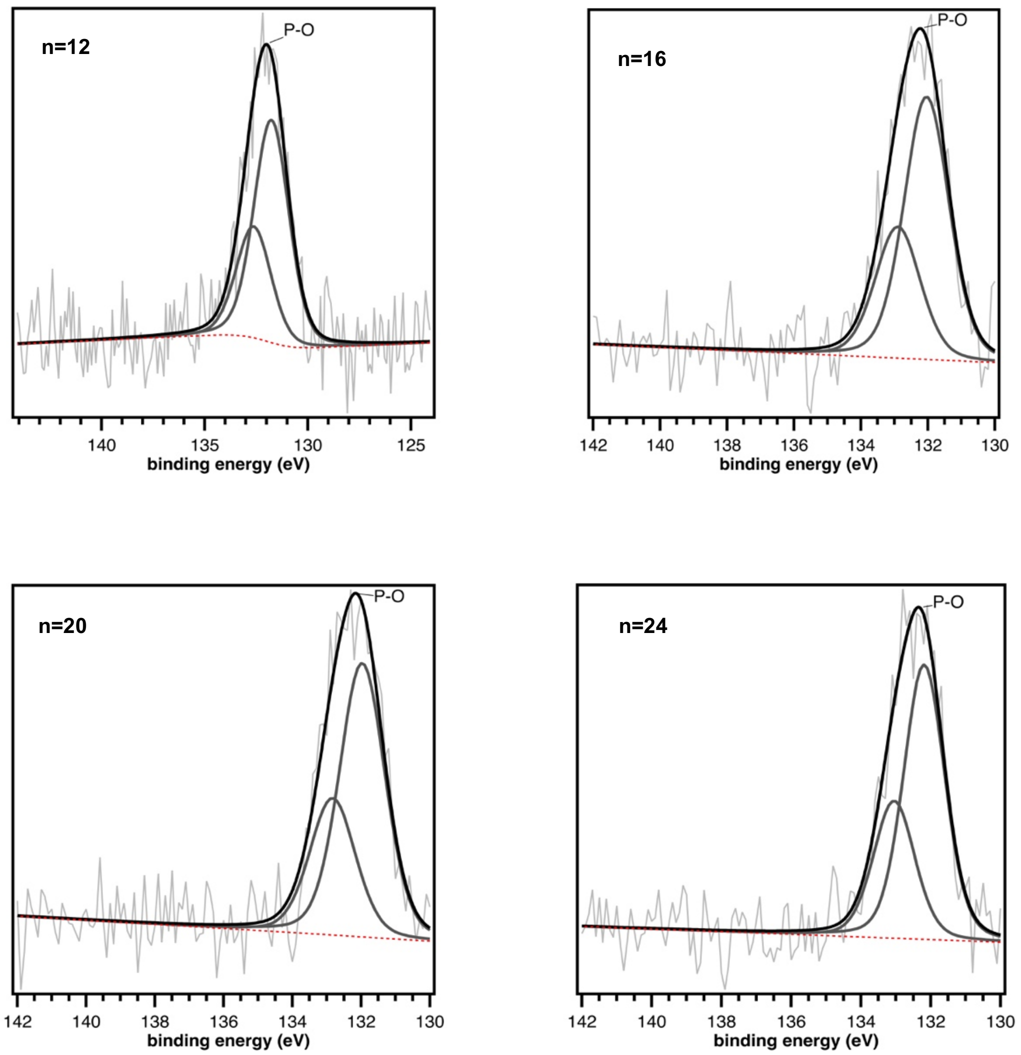

d) C1s

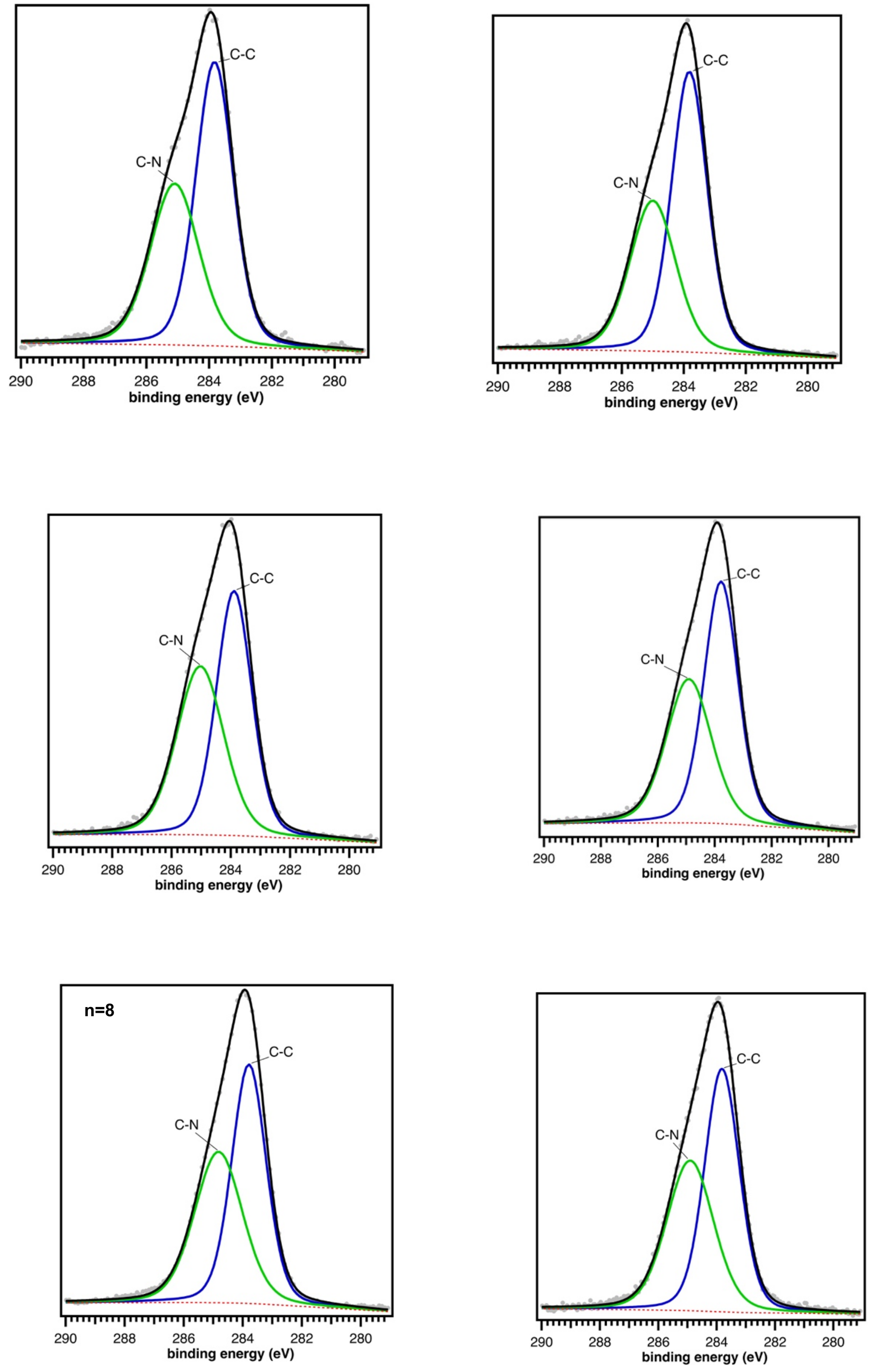

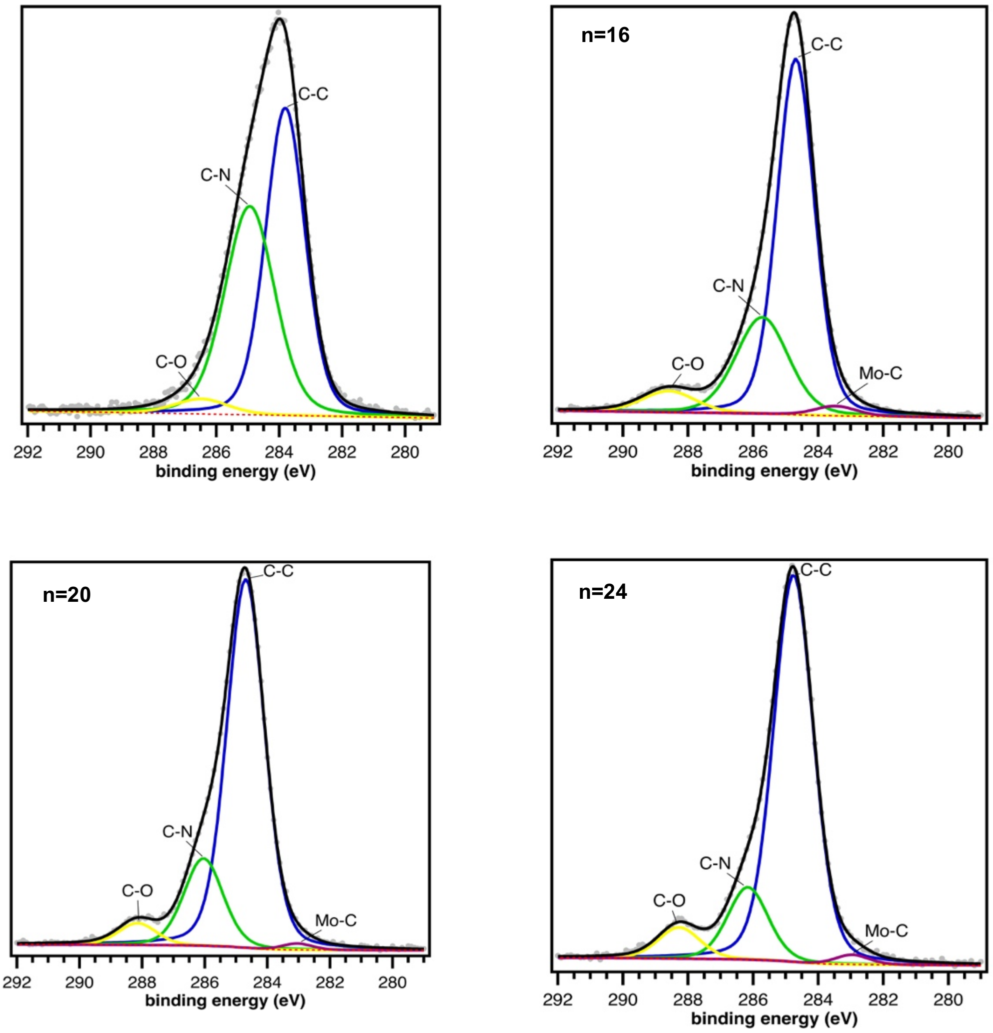
C-C
C-N
C-O
292 290 288 286 284 282 280
binding energy (eV)
n=16
C-C
C-N
C-O
Mo-C
292 290 288 286 284 282 280
binding energy (eV)
n=20
C-C
C-N
C-O
Mo-C
292 290 288 286 284 282 280
binding energy (eV)
n=24
C-C
C-N
C-O
Mo-C
292 290 288 286 284 282 280
binding energy (eV)

e) Li1s

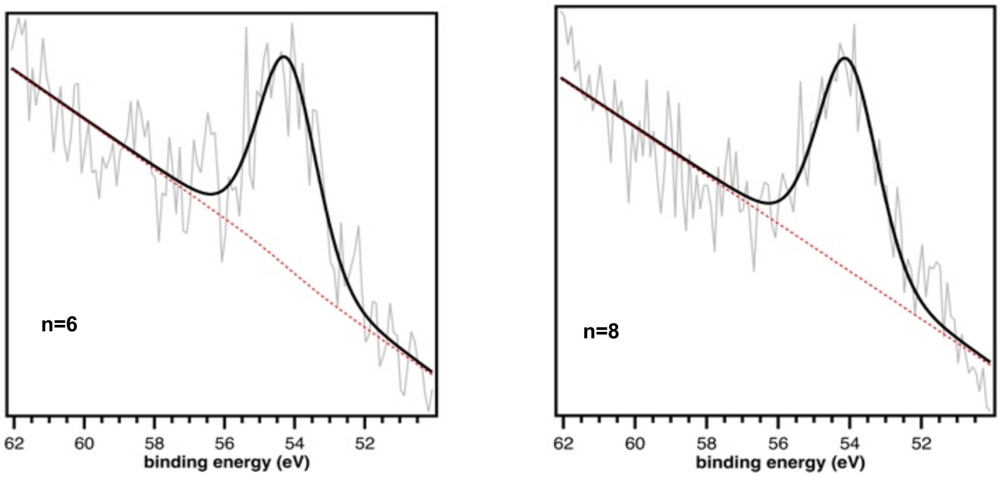
n=6
62 60 58 56 54 52
binding energy (eV)
n=8
62 60 58 56 54 52
binding energy (eV)

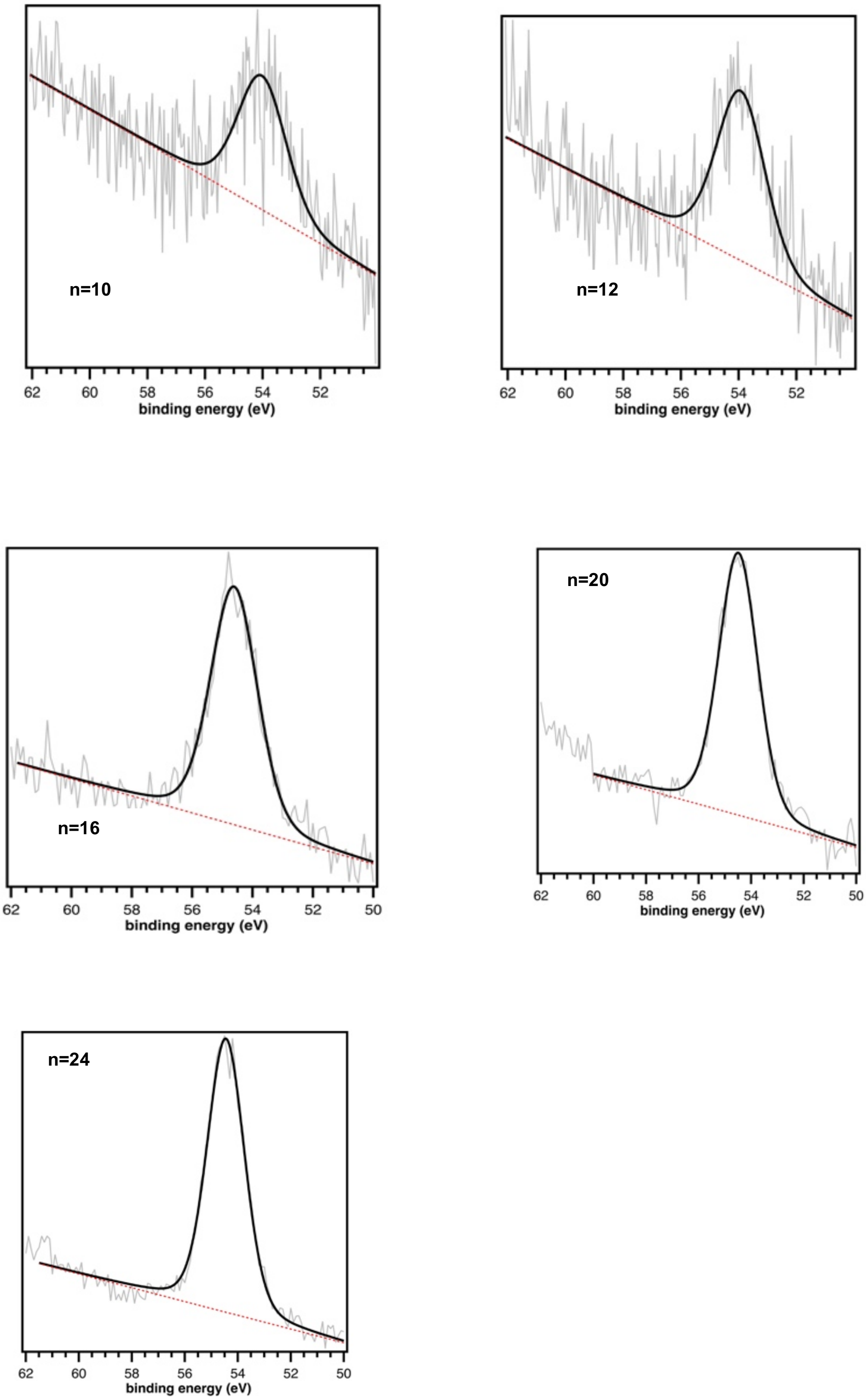

Figure S4. XPS spectra for **PMo_{12}/n** products (a) Mo3d (b) O1s (c) P2p (d) C1s (e) Li1s

Table S4. Summary of the average of Δ_{BE} for molybdenum oxides.

Compound or Functional group	Core level	ΔE (eV)	Standard Deviation	Ref.
**Mo(VI)**	Mo $3d_{5/2}$-O1s	298.0	0.3	12-17
**Mo (V)**	Mo $3d_{5/2}$-O1s	299.3	-	13
**Mo(IV)**	Mo $3d_{5/2}$-O1s	300.9	0.3	12-17
**Mo_2C**	Mo $3d_{5/2}$-C1s	54.4	0.7	18-21
**C=O**	C1s-O1s	244.25	0.2	22-23

Cyclic Voltammetry

Table S5. Reduction potentials (V) for **PMo_{12}/*n*** products vs. Ag/$AgNO_3$

Compound	$E^{A}_{1/2}$	$E^{B}_{1/2}$	$E^{C}_{1/2}$	E^{oxD}	E^{oxE}
PMo_{12}/2	-0.165	-0.379	0.679		
PMo_{12}/4	-0.150	-0.379	-0.659		
PMo_{12}/6	-0.149	-0.360	-0.657		
PMo_{12}/8	-0.149	-0.375	-0.659	0.458	
PMo_{12}/10	-0.165	-0.367	-0.654	0.454	0.967

Solid state ^{31}P NMR of soluble and insoluble components of PMo_{12}/*n* products

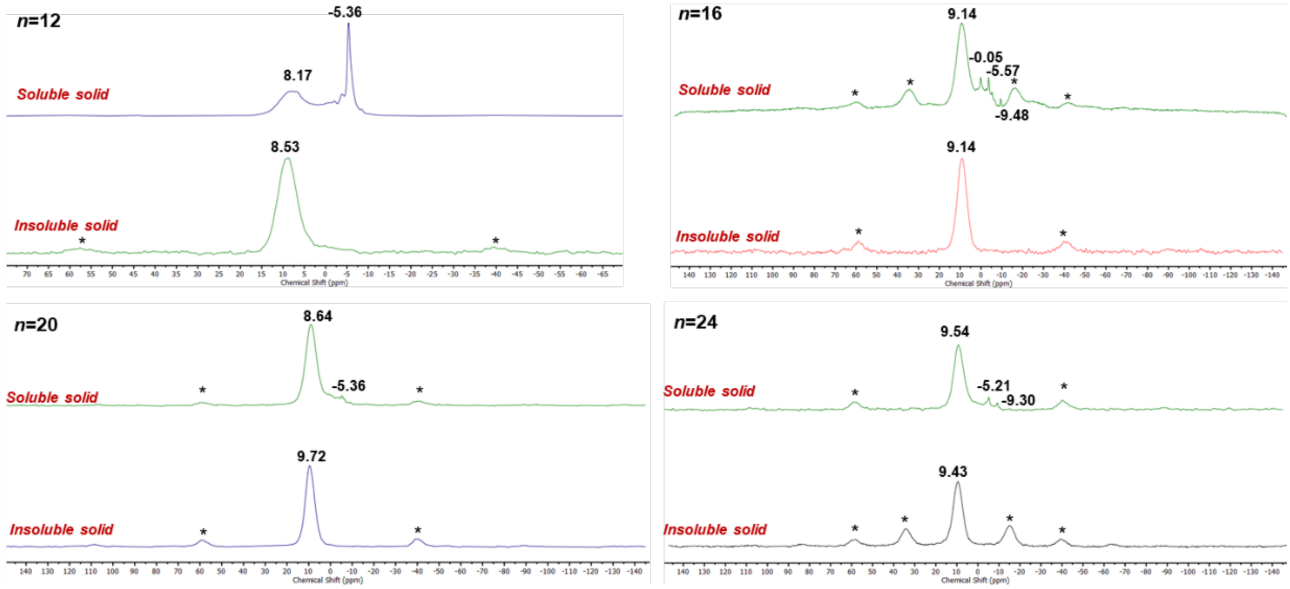

Figure S5. Solid-State ^{31}P NMR of the soluble and insoluble components obtained from **PMo_{12}/n** products (*n* = 12 – 24) after extraction into acetonitrile, filtration and evaporation of the filtrate. Peaks marked with an asterisk are spinning side-bands.

X-ray crystallographic data for $(TBA)_4[PMo_{12}O_{40}\{Li(NCMe)\}$

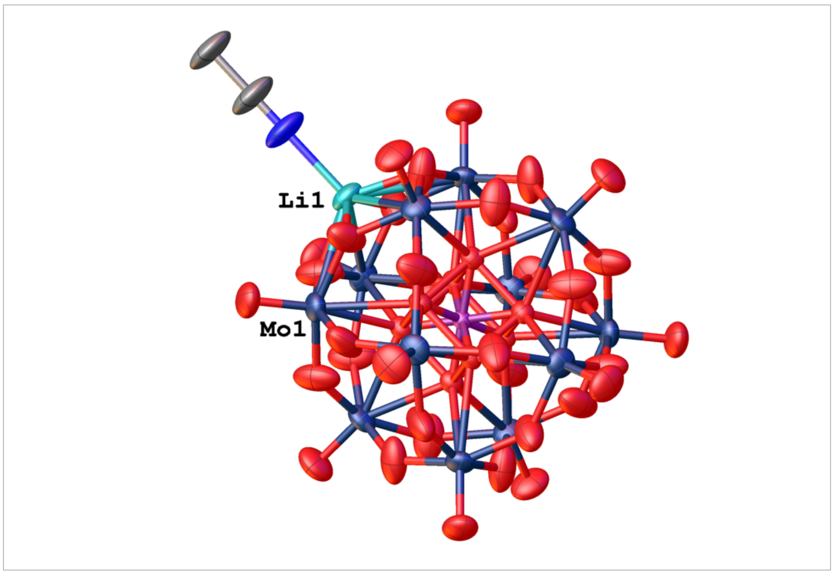

Figure S6. The anion in the structure of $(TBA)_4[PMo_{12}O_{40}\{Li(NCMe)\}]$ with ellipsoids drawn at the 50% probability level. Only one of the disorder orientation has been shown and the cations have been omitted for clarity.

Table S6. Crystal data and structure refinement for $(TBA)_4[PMo_{12}O_{40}\{Li(NCMe)\}]$.

Empirical formula	$C_{66}H_{144}LiMo_{12}N_5O_{40}P$
Formula weight	2837.04
Temperature/K	150.0(2)
Crystal system	tetragonal
Space group	I4/m
a/Å	18.7193(2)
b/Å	18.7193(2)
c/Å	14.5131(3)
α/°	90
β/°	90
γ/°	90
Volume/Å^3	5085.57(15)
Z	2
ρ_{calc}g/cm^3	1.853
μ/mm^{-1}	12.511
F(000)	2834.0
Crystal size/mm^3	0.36 × 0.08 × 0.05
Radiation	Cu Kα (λ = 1.54184)
2Θ range for data collection/°	7.708 to 133.03
Index ranges	-22 ≤ h ≤ 22, -22 ≤ k ≤ 21, -17 ≤ l ≤ 12
Reflections collected	21213
Independent reflections	2337 [R_{int} = 0.0538, R_{sigma} = 0.0238]
Data/restraints/parameters	2337/324/233
Goodness-of-fit on F^2	1.099
Final R indexes [I>=2σ (I)]	R_1 = 0.0589, wR_2 = 0.1501
Final R indexes [all data]	R_1 = 0.0650, wR_2 = 0.1554
Largest diff. peak/hole / e Å^{-3}	1.09/-1.33

Potentiometric redox titrations

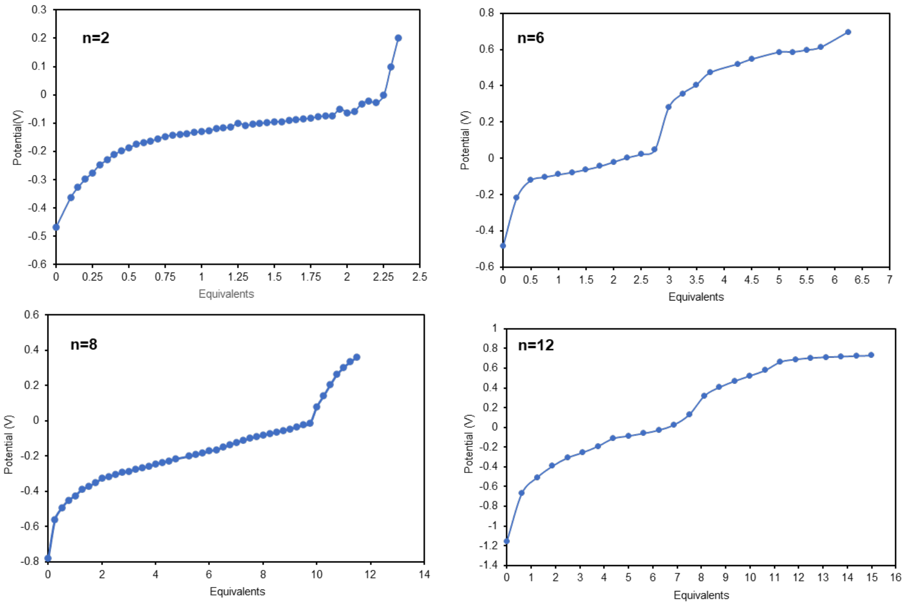

Figure S7. Titrations of **PMo$_{12}$/*n*** products in MeCN with 0.01 *M* CAN in MeCN: (a) 1.0 m*M* **PMo$_{12}$/2**, (b) 0.2 m*M* **PMo$_{12}$/6**, (c) 0.4 m*M* **PMo$_{12}$/8**, (d) 0.4 m*M* **PMo$_{12}$/12**. Formula weights used to calculate the number of equivalents were derived from idealised formulae (TBA)$_3$[PMo$_{12}$O$_{40}$Li$_n$].

Magnetic susceptibility measurements

Table S7. Magnetic Susceptibilities for **PMo$_{12}$/n** products at 25 °C.

n	Δ_v[a] (Hz)	$\Delta_{\chi M}$[b] x 10^6	μ_{exp}[c]
1[d]	6.06	964.1	1.50
2[d]	-2.19	-348.4	(-)0.91
4[d]	1.47	233.9	0.76
6[d]	1.56	248.2	0.77
8[d]	3.03	482.0	1.06
20[e]	1.92	305.4	0.78

[a] Δ_v = ^{1}H NMR chemical shift difference (Hz) for methyl protons of ^{t}BuOH in 5 - 6 m*M* solutions of **PMo$_{12}$/n** and TBA$_3$[PMo$_{12}$O$_{40}$]. [b] $\Delta_{\chi M}$ = difference in molar magnetic susceptibilities between **PMo$_{12}$/n** and TBA$_3$[PMo$_{12}$O$_{40}$]. [c] Magnetic moment calculated from molar magnetic susceptibilities obtained from the ^{1}H NMR chemical shift difference (ppm) for methyl protons of ^{t}BuOH in 5 - 6 m*M* solutions of **PMo$_{12}$/n** and TBA$_3$[PMo$_{12}$O$_{40}$]. [d] 5.00 m*M* solution. [e] 5.95 m*M* solution.

(a)

(b)

(c)

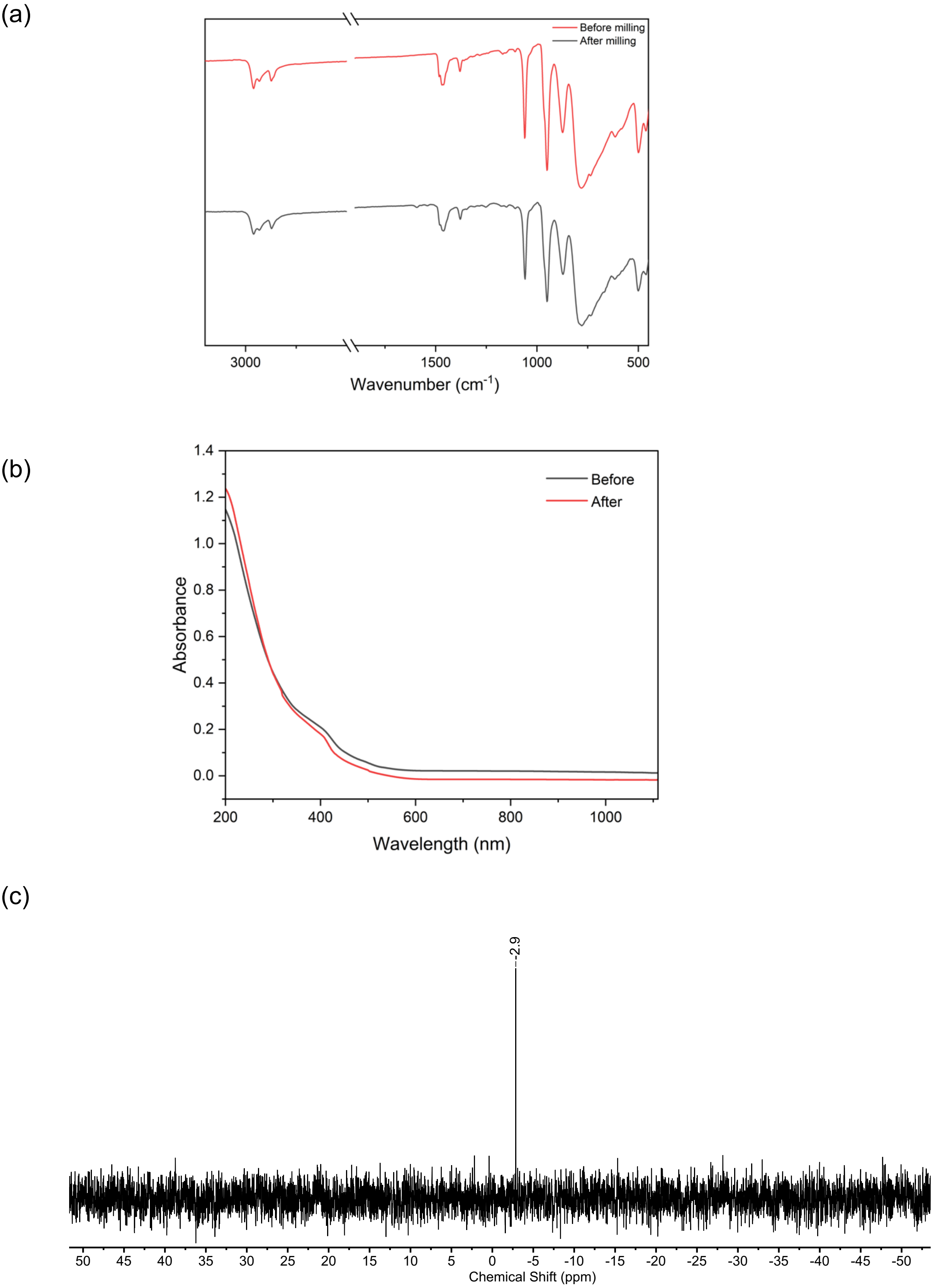

Figure S8. (a) ATR FTIR spectra and (b) UV-vis spectra of $(TBA)_3[PMo_{12}O_{40}]$ before and after ball-milling, and (c) ^{31}P NMR spectrum of ball-milled $(TBA)_3[PMo_{12}O_{40}]$.

SUPPORTING INFORMATION

References

1. Errington, R. J., *Advanced Practical Inorganic and Metalorganic Chemistry*. Blackie Academic & Professional: London, 1997.
2. Sanchez, C.; Livage, J.; Launay, J. P.; Fournier, M.; Jeannin, Y. Electron delocalization in mixed-valence molybdenum polyanions. *J. Am. Chem. Soc.* **1982,** *104*, 3194-3202.
3. Schmid, M.; Steinrück, H.-P.; Gottfried, J. M. A new asymmetric Psuedo-Voigt function for more efficient fitting of XPS lines. *Surf. Interface Anal*. **2014**, *46*, 505-511; **2015**, *47*, 1080-1080.
4. Penfold, T. J.; Tavernelli, I.; Milne, C. J.; Reinhard, M.; Nahhas, A. E.; Abela, R.; Rothlisberger, U.; Chergui, M. A wavelet analysis for the X-ray absorption spectra of molecules. *J. Chem. Phys*. **2013**, *138*, 014104.
5. Clark, R.; Reid, J. The analytical calculation of absorption in multifaceted crystals. *Sect. A: Found. Crystallogr.***1995**, *51*, 887-897.
6. CrysAlisPro, Rigaku Oxford Diffraction, Tokyo, Japan.
7. Sheldrick, G. M. SHELXT–Integrated space-group and crystal-structure determination. *Sect. A: Found. Crystallogr.* **2015**, *71*, 3-8.
8. Sheldrick, G.M. A short history of SHELX, *Acta Crystallogr., Sect. A: Found. Crystallogr.* **2008**, *64*, 112-122.
9. Dolomanov, O.V.; Bourhis, L.J.; Gildea, R.J.; Howard, J.A.K.; Puschmann, H. OLEX2: a complete structure solution, refinement and analysis program, *J. Appl. Cryst.* **2009**, *42*, 339-341.
10. Kozik, M.; Casan-Pastor, N.; Hammer, C. F.; Baker, L. C. W., Ring currents in wholly inorganic heteropoly blue complexes. Evaluation by a modification of Evans' susceptibility method. *J. Am. Chem. Soc*. **1988**, *110*, 7697-7701
11. Lin, C.-G.; Hutin, M.; Busche, C.; Bell, N. L.; Long, D.-L.; Cronin, L. Elucidating the paramagnetic interactions of an inorganic–organic hybrid radical-functionalized Mn-Anderson cluster. *Dalton Trans*. **2021**, *50*, 2350-2353.
12. Spevack, P. A.; McIntyre, N. S. Thermal reduction of molybdenum trioxide. *J. Phys. Chem* **1992,** *96*, 9029-9035.
13. Clayton, C.; Lu, Y. Electrochemical and XPS evidence of the aqueous formation of Mo_2O_5. *Surf. Interface Anal.* **1989,** *14*, 66-70.
14. Baltrusaitis, J.; Mendoza-Sanchez, B.; Fernandez, V.; Veenstra, R.; Dukstiene, N.; Roberts, A.; Fairley, N. Generalized molybdenum oxide surface chemical state XPS determination via informed amorphous sample model. *Appl. Surf. Sci.* **2015,** *326*, 151-161.
15. Patterson, T. A.; Carver, J. C.; Leyden, D. E.; Hercules, D. M. A surface study of cobalt-molybdena-alumina catalysts using x-ray photoelectron spectroscopy. *J. Phys. Chem* **1976,** *80*, 1700-1708.
16. Zingg, D.; Makovsky, L. E.; Tischer, R.; Brown, F. R.; Hercules, D. M. A surface spectroscopic study of molybdenum-alumina catalysts using X-ray photoelectron, ion-scattering, and Raman spectroscopies. *J. Phys. Chem.* **1980,** *84*, 2898-2906.
17. Sarma, D.; Rao, C. XPES studies of oxides of second-and third-row transition metals including rare earths. *J. Electron Spectrosc. Relat. Phenom.* **1980,** *20*, 25-45.
18. Ho, S.-F.; Contarini, S.; Rabalais, J. Ion-beam-induced chemical changes in the oxyanions (MO_y^{n-}) and oxides (MO_x) where M = chromium, molybdenum, tungsten, vanadium, niobium and tantalum. *J. Phys. Chem.* **1987,** *91*, 4779-4788.
19. Ramqvist, L.; Hamrin, K.; Johansson, G.; Fahlman, A.; Nordling, C. Charge transfer in transition metal carbides and related compounds studied by ESCA. *J. Phys. Chem. Solids* **1969,** *30*, 1835-1847.
20. Brainard, W. A.; Wheeler, D. R. An XPS study of the adherence of refractory carbide silicide and boride rf-sputtered wear-resistant coatings. *J. Vac. Sci. Technol.* **1978,** *15*, 1800-1805.
21. Leclercq, L.; Provost, M.; Pastor, H.; Grimblot, J.; Hardy, A.; Gengembre, L.; Leclercq, G. Catalytic properties of transition metal carbides: I. Preparation and physical characterization of bulk mixed carbides of molybdenum and tungsten. *J. Catal.* **1989,** *117*, 371-383.
22. Kundu, S.; Wang, Y.; Xia, W.; Muhler, M. Thermal stability and reducibility of oxygen-containing functional groups on multiwalled carbon nanotube surfaces: a quantitative high-resolution XPS and TPD/TPR study. *J. Phys. Chem. C* **2008,** *112*, 16869-16878.
23. Amor, S. B.; Baud, G.; Jacquet, M.; Nanse, G.; Fioux, P.; Nardin, M. XPS characterisation of plasma-treated and alumina-coated PMMA. *Appl. Surf. Sci.* **2000,** *153*, 172-183.
